# Estimating the COVID-19 infection fatality ratio accounting for seroreversion using statistical modelling

**DOI:** 10.1038/s43856-022-00106-7

**Published:** 2022-05-19

**Authors:** Nicholas F. Brazeau, Robert Verity, Sara Jenks, Han Fu, Charles Whittaker, Peter Winskill, Ilaria Dorigatti, Patrick G. T. Walker, Steven Riley, Ricardo P. Schnekenberg, Henrique Hoeltgebaum, Thomas A. Mellan, Swapnil Mishra, H. Juliette T. Unwin, Oliver J. Watson, Zulma M. Cucunubá, Marc Baguelin, Lilith Whittles, Samir Bhatt, Azra C. Ghani, Neil M. Ferguson, Lucy C. Okell

**Affiliations:** 1 MRC Centre for Global Infectious Disease Analysis; and the Abdul Latif Jameel Institute for Disease and Emergency Analytics (J-IDEA), School of Public Health, Imperial College London, London, UK; 2 Department of Clinical Biochemistry, Royal Infirmary of Edinburgh, Edinburgh, UK; 3 Nuffield Department of Clinical Neurosciences, University of Oxford, Oxford, UK; 4 Department of Mathematics, Imperial College, London, UK

**Keywords:** Respiratory tract diseases, Computational biology and bioinformatics

## Abstract

**Background:**

The infection fatality ratio (IFR) is a key statistic for estimating the burden of coronavirus disease 2019 (COVID-19) and has been continuously debated throughout the COVID-19 pandemic. The age-specific IFR can be quantified using antibody surveys to estimate total infections, but requires consideration of delay-distributions from time from infection to seroconversion, time to death, and time to seroreversion (i.e. antibody waning) alongside serologic test sensitivity and specificity. Previous IFR estimates have not fully propagated uncertainty or accounted for these potential biases, particularly seroreversion.

**Methods:**

We built a Bayesian statistical model that incorporates these factors and applied this model to simulated data and 10 serologic studies from different countries.

**Results:**

We demonstrate that seroreversion becomes a crucial factor as time accrues but is less important during first-wave, short-term dynamics. We additionally show that disaggregating surveys by regions with higher versus lower disease burden can inform serologic test specificity estimates. The overall IFR in each setting was estimated at 0.49–2.53%.

**Conclusion:**

We developed a robust statistical framework to account for full uncertainties in the parameters determining IFR. We provide code for others to apply these methods to further datasets and future epidemics.

## Plain language summary

Large-scale outbreaks of infectious diseases such as COVID-19, known as epidemics, can be monitored via statistics like the probability of death once infected, or infection fatality ratio (IFR). Measuring the levels of antibodies (proteins produced by the immune system to target the virus) in peoples’ blood can show how many have been previously infected. The number of deaths and infections are used to calculate the IFR, but this calculation is challenging due to time delays during the natural course of illness as well as imperfect antibody tests and declining antibody levels over time. We develop a mathematical model that can account for these factors to provide accurate IFR estimates. We tested our model using several different datasets. We provide code for other researchers, which can be used to obtain more accurate IFR estimates both during COVID-19 and future epidemics.

## Abstract (web summary)

Brazeau et al. use a statistical modelling approach to estimate COVID-19 infection fatality ratios from seroprevalence data. The authors’ model accounts for seroreversion over the course of the pandemic, as well as other important uncertainties such as serologic test characteristics.

## Introduction

One of the most contested statistics during the coronavirus disease 2019 (COVID-19) pandemic has been the infection fatality ratio (IFR): the proportion of those infected who will go on to die from that infection. In the first general wave of the pandemic, estimates of the overall COVID-19 IFR ranged from <0.01 to 2.3%, with a review combining estimates across studies reporting an overall estimate of 0.68% (0.53–0.82%)^1–3^. In addition, an analysis using pooled data from national serologic surveys to estimate age-specific IFRs found that the IFR rose steeply with age, ranging from <0.01% in those aged under 30 to 7.3% in the 80 and older age group^2^, broadly consistent with previous estimates^4–6^. IFRs are expected to vary across populations due to: the age distribution of the population, the distribution of infection across age groups, access to healthcare resources, the prevalence of underlying health conditions in the population, biological sex, and other factors. In addition, the overall population IFR may differ depending on the magnitude of outbreaks in care-home settings, where mortality has often been high^7^. As a result, heterogeneity is expected between locations and reflecting this variation is paramount for an accurate representation of the global COVID-19 IFR.

Estimating the IFR requires two key pieces of information: data on deaths and data on the number of infections in the population. Although there are challenges with quantifying and defining COVID-19 deaths, these data are widely reported and one of the more reliable indicators of COVID-19 burden in countries with good testing and reporting systems. However, determining the cumulative number of people infected in a population has proved to be far more challenging. Testing capacity has often been limited and many infections are asymptomatic^8^, which makes laboratory confirmed symptomatic case numbers a poor estimate of infection attack rates. As a result, serologic tests (detecting antibodies) have been used to estimate cumulative infections among populations. These tests have several limitations: (1) tests rely on a humoral immune response and will miss infections that do not mount a detectable antibody response or recent infections where antibodies have not yet developed; (2) antibodies naturally wane over time, which can lead to seroreversion (defined in this context as an individual with a confirmed infection and positive serologic test later testing negative); (3) tests will produce imperfect results (i.e. sensitivity and specificity are <100%). Many published studies reporting IFRs did not account for uncertainty in serologic test sensitivity and specificity, nor delays from onset to death and onset to seroconversion (although there are exceptions^2,9–11^) and the possibility of seroreversion has not usually been considered (again with exceptions^11^). Failing to account simultaneously for these factors could potentially lead to biased estimates of the IFR in directions that are hard to predict.

Here, we develop a novel flexible Bayesian statistical framework for estimating the IFR that accounts simultaneously for all the factors listed above. We show that accounting for these factors is critical in accurately estimating the IFR, and that seroreversion starts to significantly affect IFR estimates some months after the start of the pandemic. Similar to previous studies, we find that although overall IFR estimates vary substantially, with age-specific IFRs demonstrating a nearly log-linear pattern. From these updated calculations, we also show that early IFR estimates were relatively accurate despite not incorporating seroreversion. Our method and open-access code provide a tool for analysing IFR using further serologic datasets in the future.

## Methods

### Crude and test-adjusted IFR estimates

The crude IFR was calculated by dividing the number of observed cumulative deaths at the serologic study midpoint by the cumulative number of infections at the same time point. The number of infections was estimated as the observed seroprevalence multiplied by the population size, plus COVID-19 deaths occurring up to the midpoint of the serosurvey to avoid survival bias. The 95% confidence intervals on the crude IFR were calculated using a Monte Carlo sampling approach, where the uncertainty in the seroprevalence was propagated by drawing 100,000 values of the expected seroprevalence based on the binomial distribution (i.e. the number of test-positives given the total tested). For Denmark, Italy, and Sweden where only the seroprevalence and confidence intervals were reported (i.e. counts of test-positives and total tested were not available) intervals were logit-transformed and used to calculate variances directly. Test-adjusted simple IFR estimates were calculated in the same way, but first adjusting the seroprevalence for the sensitivity and specificity of the serologic test used in the study^12^.

### Statistical model for estimating IFR

#### Daily and age-stratified deaths

For individuals who die following infection, we assume that the time from infection to death follows a gamma distribution with shape $\alpha$ and rate $\beta$. If an individual is infected at time $t$ then the probability that they die at time ${t}_{d}$ is:

1 $${{{{{\rm{Pr }}}}}}\left({t}_{d}\,|t,\alpha ,\beta \right)\,=\,\frac{{\beta }^{\alpha }}{\Gamma \left(\alpha \right)}{({t}_{d}\,-\,t)}^{\alpha \,-\,1}{e}^{-\beta ({t}_{d}\,-\,t)}$$

We make the simplifying assumption that time is discrete and measured in days, defining $\tau ({T|t})$ to be the probability of death on day $T\,\in\, {Z}_{ > 0}$ given infection at the start of day $t\,\in\, {Z}_{ > 0}$ where $t\,\le\, T$:

2 $$\tau \left(T|t\right)\,=\, \int _{T\,-\,1}^{T}\frac{{\beta }^{\alpha }}{\Gamma \left(\alpha \right)}{({t}_{d}\,-\,t\,+\,1)}^{\alpha \,-\,1}{e}^{-\beta ({t}_{d}\,-\,t\,+\,1)}{{dt}}_{d}$$

(the $+1$ term in the above comes about because we assume infections occur at the start of the day, but deaths can be registered until the end of the day, hence $\tau \left(1\right)$ returns a positive value).

Our population is split into different age strata, each with their own probabilities of infection and death. Let there be $A\in {Z}_{ > 0}$ age groups in total, and let ${p}_{a}$ be the proportion of the total population in age group $a\in 1:A$. In the simplest model we would expect infections to occur in a given age group in proportion to the number of people in that group. To allow for variation in age-specific attack rates, and in order to fit to age-specific seroprevalence data, we include a multiplicative attack rate scalar ${k}_{a}$ within each group, allowing the final attack rate to be higher or lower than expected from proportions alone. Hence the overall probability of infection in age group $a$, which will be written ${\rho }_{a}$, is given by:

3 $${\rho }_{a}\,=\,\frac{{\rho }_{a}{k}_{a}}{{\sum }_{i\,=\,1}^{A}{p}_{i}{k}_{i}}$$

Once infected, the probability of death in age group $a$ (i.e. the IFR in this age group) is defined as ${m}_{a}$. Hence, the overall probability of an individual in age group $a$ dying on day $T$ given infection on day $t$ can be written ${\rho }_{a}{m}_{a}\tau ({T|t})$.

Our raw data do not consist of individual-level outcomes, but rather aggregate counts. Specifically, two marginal distributions were available for each study: (1) daily counts of the number of COVID-19 deaths, summed over all age groups, and (2) the cumulative number of COVID-19 deaths at a single point in time, but broken down by age. Both marginal distributions were fit within a single statistical framework.

Let ${I}_{t}$ be the number of new SARS-CoV-2 infections in the population on day $t$. The true infections curve is unknown, and was modelled using an exponentiated natural cubic spline, subject to the constraint that the total number infected *(*i.e. the area under the curve) could not exceed the total population size $N$. It follows from the definitions above that the number of infections in age group $a$ on day $t$ is given by ${\rho }_{a}{I}_{t}$, and the number of ultimately fatal infections is given by ${\rho }_{a}{{m}_{a}I}_{t}$. The expected total deaths on day $T$, denoted ${\mu }_{T}$, is obtained by summing over all age groups and all possible times of infection as follows:

4 $${{{\upmu }}}_{T}\,=\,\mathop{\sum }\limits_{a\,=\,1}^{A}{\rho }_{a}{m}_{a}\mathop{\sum }\limits_{t\,=\,1}^{T}{I}_{t}\tau \left(T|t\right)$$

The observed number of COVID-19 deaths on day $T$, denoted ${D}_{T}$, is assumed to be Poisson distributed around this expectation:

5 $${{{{{\rm{Pr }}}}}}\left({D}_{T}\,|\,{{{\upmu }}}_{T}\right)\,=\,\frac{{({{{\upmu }}}_{T})}^{{D}_{T}}{e}^{-{{{\upmu }}}_{T}}}{{D}_{T}!}$$

The likelihood for this part of the model is simply the product of Poisson probabilities over all days in our time series:

6 $${{L}}_{1}\,=\,\mathop{\prod}\limits_{T}{{{{{\rm{Pr }}}}}}({D}_{T}\,|{{{\upmu }}}_{T})$$

Moving on to the second marginal distribution, the expected cumulative deaths in age group $a$ up until time $Y$ can be written:

7 $${q}_{a}\,=\,{\rho }_{a}{m}_{a}\mathop{\sum }\limits_{T\,=\,1}^{Y}\mathop{\sum }\limits_{t\,=\,1}^{T}{I}_{t}\tau \left(T|t\right)$$

These expected values are converted into expected proportions of deaths in each age group as follows:

8 $${f}_{a}\,=\,\frac{{q}_{a}}{{\sum }_{i\,=\,1}^{A}{q}_{i}}$$

Finally, the observed cumulative COVID-19 deaths up until day $Y$, denoted by the vector $C$ with elements ${C}_{a}$ for $a\,\in\, 1:A$, are assumed to be multinomially distributed with these proportions:

9 $${{{{{\rm{Pr}}}}}}(C\,|\,f)\,=\,\left(\mathop{\sum }\limits_{a\,=\,1}^{A}{C}_{a}\right)!\mathop{\prod }\limits_{a\,=\,1}^{A}\frac{{f}_{a}^{{C}_{a}}}{{C}_{a}!}$$

This is the second component of the likelihood:

10 $${{L}}_{2}\,=\,{{{{{\rm{Pr}}}}}}(C\,|\,f)$$

#### Incorporating serology data

The third data type used in fitting comes from serological studies. For a given individual infected on day $t$ we model the probability of having seroconverted by day ${t}_{s}$ using the following formula:

11 $${{{{{\rm{Pr }}}}}}\left(X\,=\,1\,|{t}_{s},t,{{\uplambda }}\right)\,=\,1\,-\,{\exp }\left(\frac{-\left({t}_{s}\,-\,t\right)}{{{\uplambda }}}\right)$$

where $X$ is a binary variable that equals 1 if the individual has seroconverted and 0 otherwise. This is equivalent to assuming seroconversion with a constant hazard $1/\lambda$. Translating to the population level, the expected number of people to have seroconverted by time $T$ in age group $a$, denoted ${\theta }_{T,a}$, is given by:

12 $${\theta }_{T,a}\,=\,{\rho }_{a}\mathop{\sum }\limits_{t\,=\,1}^{T}{I}_{t}{{{{{\rm{Pr }}}}}}\left(X\,=\,1\,|T,\,t,\,{{\uplambda }}\right)$$

This can be translated to an expected proportion via the expression ${\theta }_{T,a}/{N}_{a}$, where ${N}_{a}$ is the total population size in age group $a$, such that ${\sum }_{a\,=\,1}^{A}{N}_{a}\,=\,N$.

The observed prevalence of seropositive individuals (the seroprevalence) is expected to deviate from this proportion due to both sampling effects and imperfect test characteristics. If $\gamma \,\in\, [{{{{\mathrm{0,1}}}}}]$ is the sensitivity of the test directly after seroconversion, before antibody waning, and $\delta \,\in\, [{{{{\mathrm{0,1}}}}}]$ is the specificity then the test-adjusted expected seroprevalence, ${\phi }_{T,a}$, can be calculated using the classic Rogan-Gladen correction^12^:

13 $${{{\phi }}}_{T,a}\,=\,{{\upgamma }}\left(\frac{{\theta }_{T,a}}{{N}_{a}}\right)\,+\,\left(1\,-\,{{\updelta }}\right)\left(1\,-\,\frac{{\theta }_{T,a}}{{N}_{a}}\right)$$

Let the total number of people tested on day $T$ in age group $a$ be denoted ${s}_{T,a}$, and let the observed number of seropositives be denoted ${n}_{T,a}$. We model the observed counts as binomially distributed around the Rogan-Gladen-corrected proportion:

14 $${{\Pr }}\big({n}_{T,a}\big|{{{\phi }}}_{T,a}\big)=\left(\begin{array}{c}{{s}_{T,a}}\\ {{n}_{T,a}}\end{array}\right){{{\phi }}}_{T,a}^{{n}_{T,a}}{(1-{{{\phi }}}_{T,a})}^{{s}_{T,a}-{n}_{T,a}}$$

Finally, the likelihood for this component of the model is the product of the binomial probability over all age groups, and over all serology study dates ${T}_{y}$:

15 $${{L}}_{3}\,=\,\mathop{\prod}\limits_{y}\mathop{\prod }\limits_{a\,=\,1}^{A}{{{{{\rm{Pr }}}}}}\left({n}_{{T}_{y},a}\,|\,{{{\phi }}}_{{T}_{y},a}\right)$$

The full likelihood is the product of the individual likelihood components listed above.

#### Extension for seroreversion

As part of a sensitivity analysis, we allowed for individuals to serorevert over time under an assumption of natural waning antibodies. We assumed that individuals experience a constant hazard $1/\lambda$ of seroconverting, followed by a probability of seroreverting characterised by a Weibull distribution with shape $\kappa$ and scale $\mu$. Under these conditions, the probability of being seropositive by the end of day ${t}_{s}$ following infection on day $t$ is given by:

16 $${{{{{\rm{Pr }}}}}}\left(X\,=\,1\,|{t}_{s},t,{{\uplambda }},{{\upmu }}\right)\,=\, \int _{t}^{{t}_{s}}\frac{1}{{{\uplambda }}}{{{{{\rm{exp }}}}}}\left(-\left(\frac{x\,-\,t}{{{\uplambda }}}\right)\,-\,{\left(\frac{{t}_{s}\,-\,x}{{{\upmu }}}\right)}^{\kappa }\right){dx}$$

All subsequent steps are identical to those described above in Eqs. (12–15), resulting in an alternative version of the likelihood component ${L}_{3}$.

### Model fitting

We used informative priors for key parameters where they were well characterised, such as the delay from symptom onset to death. We fit the model using Metropolis-Coupled Markov Chain Monte Carlo (MC^3^) using the *drjacoby* R package (version 1.2.0)^13^. Full details of priors and model fitting are provided in Supplementary Table 1 and Supplementary Methods.

We re-estimated test specificity for serologic studies where regional data were available, by fitting a simplified version of the main model described above to seroprevalence and cumulative regional deaths at the midpoint of the most recent serosurvey, adjusting for age demographic differences within regions using RStan^14^ (Supplementary Methods). These estimates were then used as informative priors for the subsequent IFR analyses of each survey.

Convergence of models was assessed by visualising the posterior distributions as well as requiring the Gelman-Rubin’s convergence diagnostic to be lower than 1.1^15^. For the IFR model using MC^3^, the metropolis coupling acceptance rate between rungs was also examined.

### Application to first-wave data

To estimate the time of seroreversion after symptom onset from longitudinal serology data (see above), we fit a Weibull survival model using interval censoring to account for the uncertainty in the observed time of seroreversion. As a comparison to our parametric fit, we also fit a Kaplan–Meier survival curve with interval censoring. Models were fit using the ‘survival’ R-package^16,17^. The ‘survminer’ R-package was used in plotting the Kaplan–Meier survival curve^18^ (Supplementary Methods).

Serologic studies were selected from an existing, continuously updated systematic review: the ‘SeroTracker’ dashboard^19^. Estimates of the sensitivity and specificity of the serologic assay were obtained preferentially from validation conducted as part of each serosurvey, rather than external validation (e.g. by manufacturers). We preferentially obtained data on COVID-19 deaths by age and date of death from Ministries of Health and national public health agencies (Supplementary Table 2), and when otherwise not available, used data from the COVID-19 Data Repository by the Centre for Systems Science and Engineering at Johns Hopkins University (JHU CSSE COVID-19 Data) up to August 17, 2020 (accessed September 14, 2020)^20,21^. Similarly, demographic information was extracted from both governmental and non-governmental websites. Ethical approval was not required because the data were publicly available. Datasets are archived on Github^22^.

We calculated pooled-IFR estimates using a weighted log-linear regression on the age-specific IFR posterior estimates. Weights were incorporated as the precision from the age-specific 95% credible intervals. Prediction intervals were calculated from the log-normal density function using the mean from the model fit and model variance. Overall pooled-IFR estimates were calculated by standardising to the demographics of representative countries within the low-income country (LIC), low-middle income country (LMIC), upper-middle income country (UMIC), and high-income country (HIC) bracket, respectively^23^ (Supplementary Note 4).

### Reporting summary

Further information on research design is available in the Nature Research Reporting Summary linked to this article.

## Results

### Statistical framework and model overview

We constructed a Bayesian statistical model to estimate IFR, incorporating a number of key factors that can bias estimates away from the true value, including: (1) the delay between infection and death, (2) the dynamical process of seroconversion and seroreversion, (3) differences in age-specific attack rates, and (4) serologic test characteristics. This approach allows for full propagation of uncertainty in all these factors. The full mathematical details and model fitting process are available in Methods and Supplementary Methods. In brief, the model assumes that the observed COVID-19 daily deaths are the result of infections at an earlier point in time. This infection curve was estimated using an exponentiated natural cubic spline, and projected forwards by an infection-to-death delay distribution and age-specific IFR when fitting to daily death data. The area under the infection curve, equivalent to the cumulative incidence of infections, was then fit to the age-specific seroprevalence data at the time of each serosurvey. The model assumes that the temporal profile of the infection incidence curve is the same for all age groups but that total cumulative incidence per person can vary by age. The model also assumes constant IFR over time. We included seroreversion by assuming a distribution of times from the time of seroconversion until becoming antibody negative, estimated from published longitudinal antibody data in non-hospitalised cases (see below). We included serologic test sensitivity and specificity as parameters to be estimated in the model, using informative priors based on validation studies.

The code for reproducing these results are available as a R Research Compendium on Github: ‘mrc-ide/reestimate_covidIFR_analysis’. The IFR statistical model is available as a standalone R-package on Github: ‘mrc-ide/COVIDCurve’ (v0.5.0)^22^.

### Application to simulated data

#### Comparison of model-estimated IFR to simple IFR calculations

**Fig. 1:**
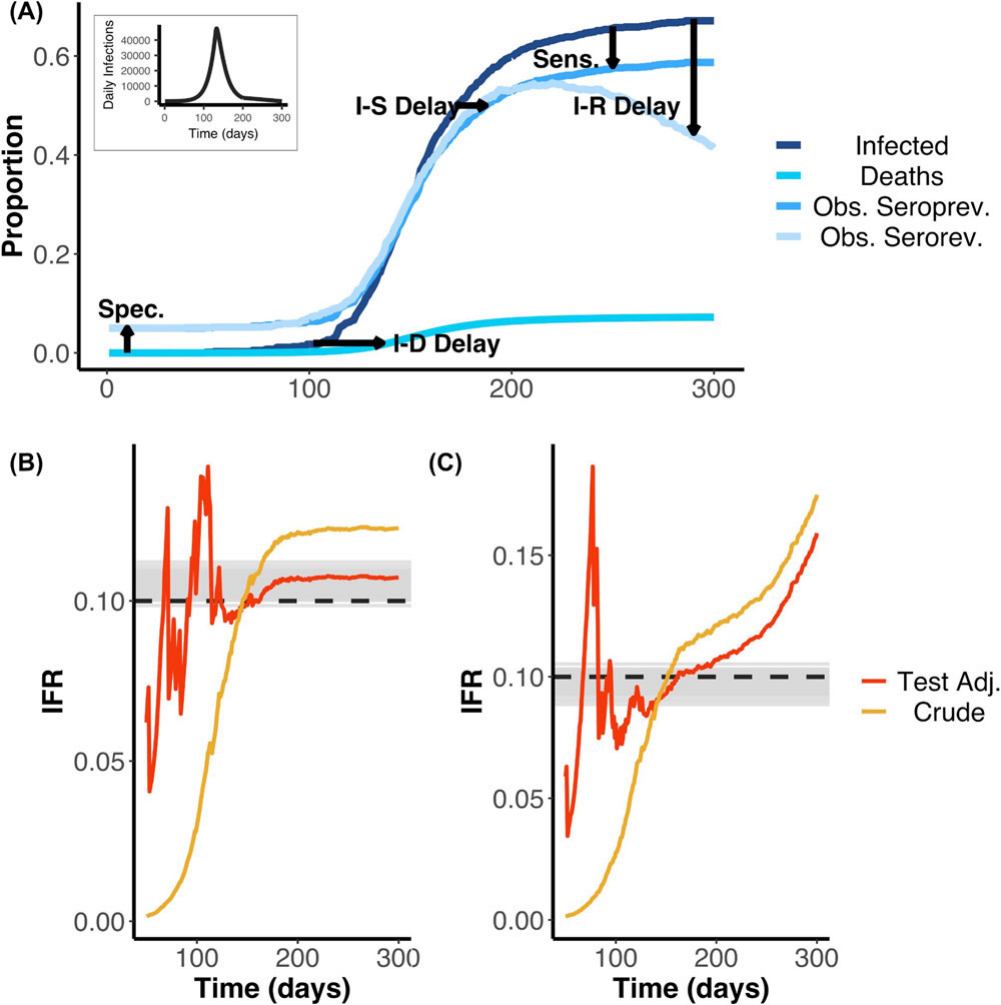
IFR estimates from serologic data. **A** Schematic showing cumulative infections, deaths and seroprevalence with and without seroreversion over time. We highlight the effects of delays from infection to seroconversion (I–S Delay), to death (I–D Delay), and to seroreversion (I–R Delay) as well as serologic test sensitivity (Sens.), serologic test specificity (Spec.) on the observed data. The daily infection curve used as input for the simulation is shown as the plot inset. Early in the outbreak, false positives dominate due to low prevalence and imperfect specificity, whilst later the difference between true cumulative incidence and observed seroprevalence is mainly due to low sensitivity and/or seroreversion. The delays show how the cumulative infection curve is lagged behind the observed seroprevalence. Similarly, the contrast of the seroprevalence curve with (Obs serorev) and without (Obs seroprev) seroreversion reveals the loss of sensitivity over time. These simulations were used as the inputs for the results displayed in (**B**, **C**). We used 0.1% of the simulated data at random (i.e. we do not assume we observe the entire population through time). **B** Estimated IFR over time based on a simulated epidemic that does not include seroreversion. Here, the simulated IFR value is indicated by the dashed black line and the grey lines indicate 100 posterior draws from the fitted statistical model (based on the posterior probability), indicating the capacity for our model framework to correctly recover the true IFR. Red and yellow lines represent the simple and test-adjusted (Rogan-Gladen correction) IFR estimates (see Main Text), calculated as if the serosurvey had been conducted on each respective day (after day 50). In the case without seroreversion, the IFR appears to be adequately captured by the Rogan-Gladen correction once infections have stopped accruing (the realised IFR appears to be slightly greater than the initial simulated true IFR value of 0.1). **C** As for (**B**), but the simulation and statistical model both include seroreversion. The IFR values are shown as a probability. In the case that includes seroreversion, the Rogan-Gladen correction can no longer adequately capture the IFR value, as seroprevalence estimates are constantly changing. In addition, in the outbreak, when the true seroprevalence is less than the false positive rate, adjusting for the serologic test characteristics can result in unstable IFR estimates.

The simplest IFR calculation takes the total number of deaths up to a given time and divides by the number of infected individuals, estimated as the percentage of seropositive individuals multiplied by the population size. We used simulations to understand how the delay from infection to outcomes and serologic test characteristics can bias the simple, crude IFR compared with the true, simulated IFR (Fig. 1). From simulations assuming no seroreversion, we found that the crude IFR tended to underestimate the true IFR when the epidemic was growing, or overestimate the true IFR when the epidemic was contracting (Fig. 1B). Moreover, even after adjusting the IFR for test performance using the Rogan-Gladen correction, a common approach to adjust for test sensitivity and specificity^12^, the true IFR was only captured when the epidemic was nearly over (Fig. 1B). These biases result from failing to account for the delays from onset of infection to death and seroconversion. When including seroreversion in the simulation, both the crude IFR and the test-adjusted IFR increasingly overestimated the true IFR as more time passed since the first wave of the epidemic (Fig. 1C). This underestimation is expected, since declining seroprevalence deflates the IFR denominator (i.e. total number infected) while the numerator (i.e. cumulative deaths) remains constant or increases. By contrast, our statistical model was able to recover the true IFR accurately when analysing simulated epidemics with and without seroreversion (Fig. 1B, C).

#### The statistical model is robust to different epidemic shapes and seroreversion

**Fig. 2:**
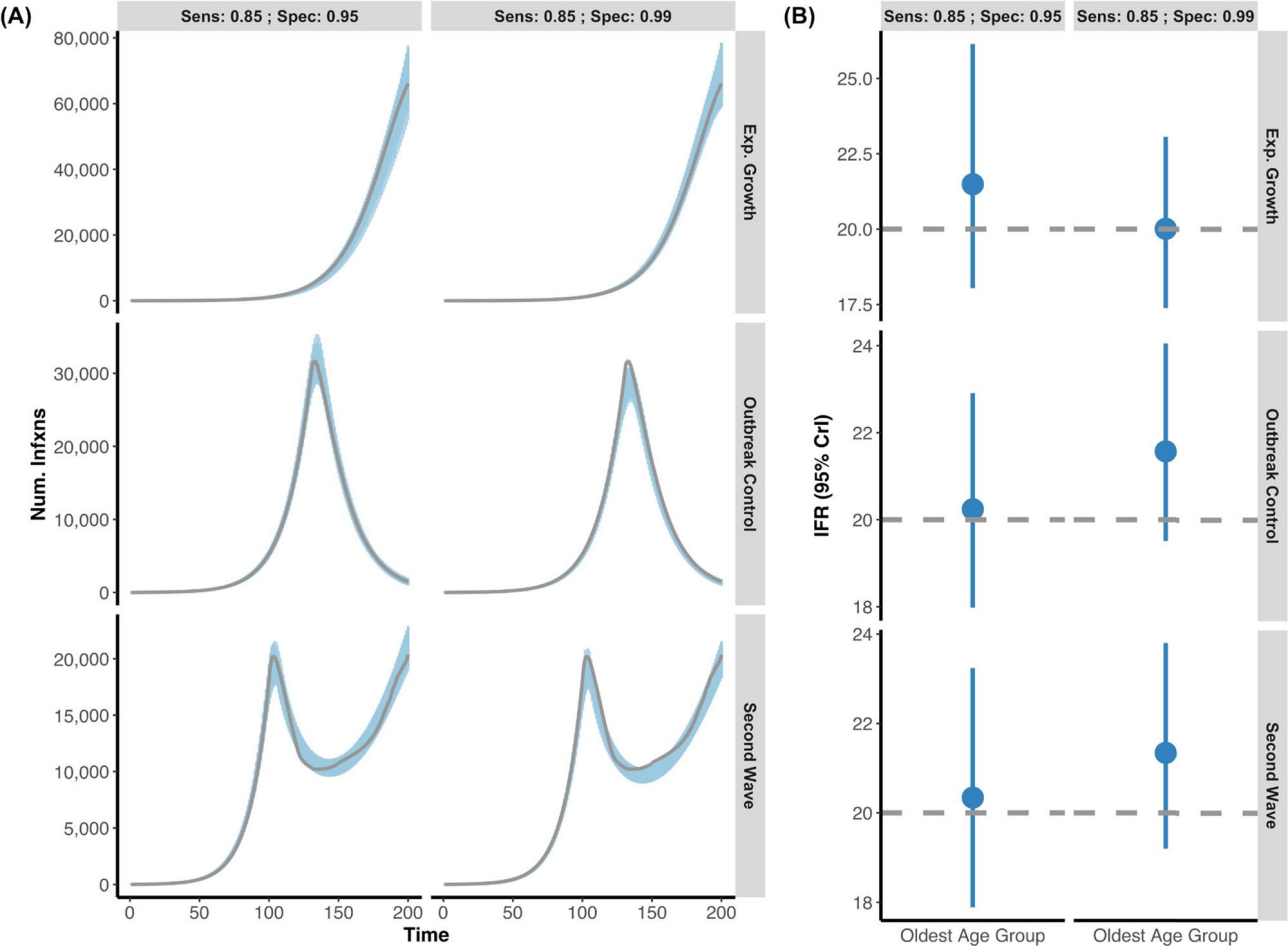
Posterior daily infections and IFR estimates from simulated data without seroreversion. **A** Using simulated data, we created three outbreak scenarios where individuals who seroconverted could not serorevert: exponential growth, outbreak control, and second wave (grey lines are simulated infection input) under two different serologic tests (Sensitivity: 85%; Specificity 95% vs. Sensitivity: 85%; Specificity 99%). The blue shading represents 100 posterior draws of the modelled infection curve, where draws were selected based on their posterior probability. **B** The inferred median and 95% credible intervals (blue) versus the simulated true IFR (grey, dashed line) with two different serologic tests, in the oldest age group. For all epidemic scenarios considered, we assume that there are two seroprevalence surveys that range over days 120–130 and 170–180 and that 0.1% of the population was sampled.

**Fig. 3:**
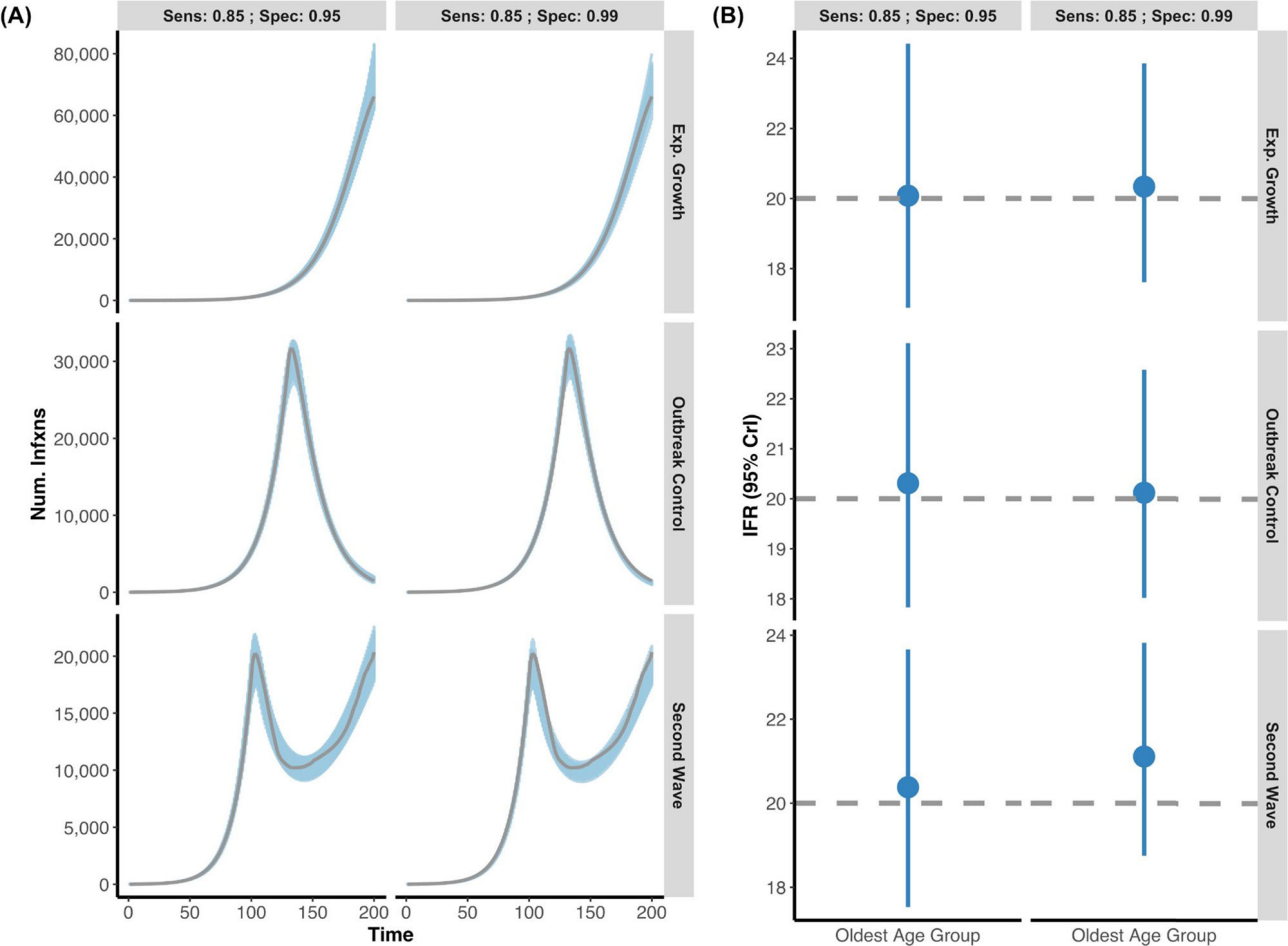
Posterior daily infections and IFR estimates from simulated data with seroreversion. **A** Three simulated epidemics were generated (exponential growth, outbreak control, and second wave) as in Fig. 2, but now with the additional feature that individuals who seroconverted would eventually serorevert. Grey lines indicate the simulated true infection curve under two different serologic tests (Sensitivity: 85%; Specificity 95% vs. Sensitivity: 85%; Specificity 99%). The blue shading represents 100 posterior draws (based on the posterior probability) of the modelled infection curve (using an exponentiated natural cubic spline), where draws were selected based on their posterior probability. **B** The inferred median and 95% credible intervals (blue) versus the simulated true IFR (grey, dashed line) in the oldest age group for each of the outbreak scenarios with respect to the two different serologic test characteristics. As above, the model accurately captures both the simulated infection curve and the simulated IFR while accounting for seroreversion. For all epidemic scenarios considered, we assume that there are two seroprevalence surveys that range over days 120–130 and 170–180 and that 0.1% of the population was sampled.

We next assessed whether our model could accurately infer IFR from epidemics with different shapes. Infection curves were simulated with exponential (unmitigated) growth, exponential growth followed by interventions that led to resolution of the outbreak, and epidemics with two waves (respectively referred to as the Exponential Growth, Outbreak Control, and Second Wave). In simulations we assumed two serosurveys were conducted over days 120–130 and 170–180. The model was able to capture the simulated true IFR value within the 95% credible interval in all scenarios, when seroreversion was and was not considered (Figs. 2, 3). In some instances, the model very slightly overestimated the simulated true IFR in the younger age group (e.g. 0.11–0.14% instead of 0.1%) whilst the older age groups’ true IFR was always captured by the 95% credible intervals. This is presumably due to the fewer deaths in the younger age groups. Additionally, our model remains able to capture the true underlying IFR in simulated data when only a single serosurvey is conducted (Supplementary Fig. 1). Uncertainty in the IFR estimates is appropriately propagated and increases when only one survey is available.

#### Serologic test specificity can be informed by analysing serosurveys by region

**Fig. 4:**
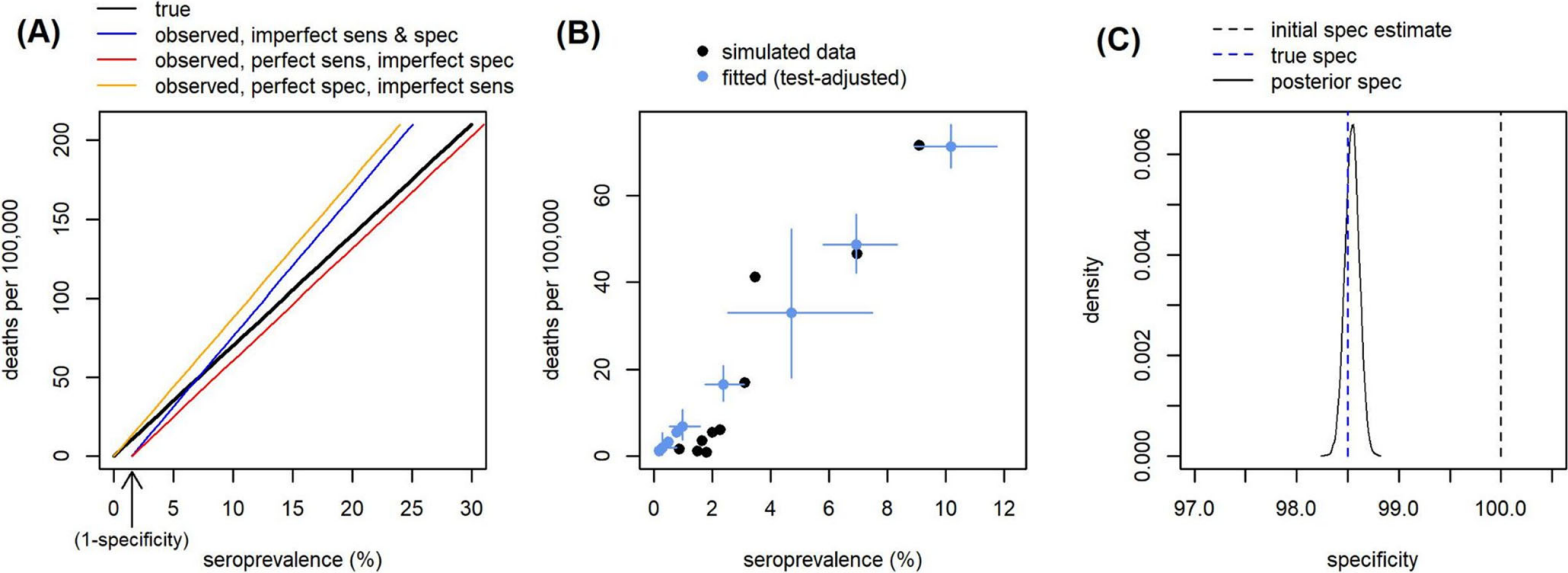
Estimating serologic test specificity from regional data. **A** Expected relationship between seroprevalence and deaths per 100,000 under different values of serologic test sensitivity and specificity, when overall IFR = 0.7% and both IFR and population age structure are constant. **B** Example simulation of seroprevalence and deaths per 100,000 in different regions within a serosurvey (black), assuming varying burden of COVID-19 and population sizes between regions, but constant test performance and IFR. Model-estimated mortality and seroprevalence (adjusted for test performance) for each region when fitting to the simulated data (blue; error bars = 95% CrI). Serologic test performance is simultaneously estimated by the model, using informative priors from a simulated validation study and the relationship between seroprevalence and mortality. **C** Initial prior specificity estimate based on a simulated validation study including 100 true negative cases (black dashed line); by chance 100% specificity was measured in the simulated validation study, although the true value is 98.5% (blue dashed line). The model fitted to simulated regional data is able to infer a much more accurate posterior specificity estimate (black solid line shows posterior distribution).

Correctly estimating serologic test specificity is critical for accurately estimating IFR, particularly when seroprevalence is low. Even an error of 1–2% in the specificity value can have a substantial impact on IFR estimates^24^. However serologic test validation studies are often based on relatively small samples which can give misleading estimates by chance. For example, if 100 negative controls are used to measure test specificity, there is a >20% chance of the test identifying all of these as negative, even if the true specificity is only 98.5%. In large serosurveys where seroprevalence varies across different regions within the survey, serologic test specificity can be additionally informed based on the relationship between seroprevalence and regional COVID-19 mortality. We generated the expected relationship for the simplest case, where the IFR is constant in each region (Fig. 4A). Seroprevalence and COVID-19 mortality are expected to have a linear relationship in which the observed seroprevalence at zero deaths and zero infections captures the false positive rate of the test (1-specificity). We simulated epidemics with varying COVID-19 burdens across regions, with 0.1–10% of the population infected (4B), and estimated test specificity from these data, using simulated validation studies as priors (Methods). We show that our analysis recovers a more accurate estimate of test specificity using regional data even when the validation study has by chance generated an inaccurate result (4C).

### Application to observed first-wave data

#### Time to seroreversion

**Fig. 5:**
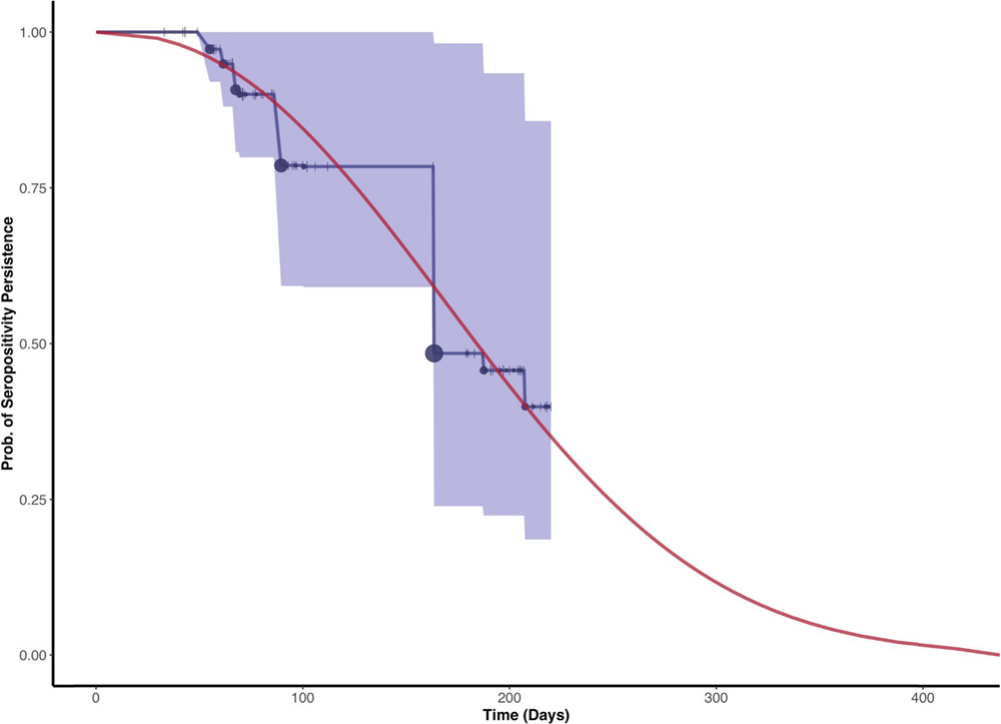
Seroreversion data and model fit. Persistence of seropositive test results with the Abbott assay among an extended cohort of 101 COVID-19 patients (extended dataset based from Muecksch et al.^25^). The interval-censored Kaplan–Meier survival curve with 95% confidence intervals (blue) with censored observations (ticks) and seroreversion events (circles) is shown for comparison. Both censoring (range 1–4) and seroreversion events (range 0–16.16) are scaled according to the number of observations on the given day. The fitted Weibull survival function (red) of persistence of a serologic positive result is shown in red. The fit was estimated from symptom onset to time of seroreversion, where the time of seroreversion was estimated incorporating interval censoring. The mean time from symptom onset to seroreversion was 190.93 days.

To estimate a realistic distribution of times to seroreversion, we used an extended set of published of longitudinal SARS-CoV-2 IgG N-antibody assay data collected for ~5.5–7.5 months among non-hospitalised participants with real-time PCR-confirmed SARS-CoV-2 infections^25^. We fit a survival model to these data and found the times to seroreversion could be well characterised by a Weibull distribution. The mean time from symptom onset to seroreversion was 190.93 days, well within first-wave timeframes (Fig. 5, Weibull shape parameter: 2.32; Weibull scale parameter 215.50). We selected Abbott assay data as it demonstrated the greatest loss in sensitivity over time (i.e. the most seroreversion), so as to look at a maximal effect of seroreversion on IFR estimates.

#### First-wave data

**Fig. 6:**
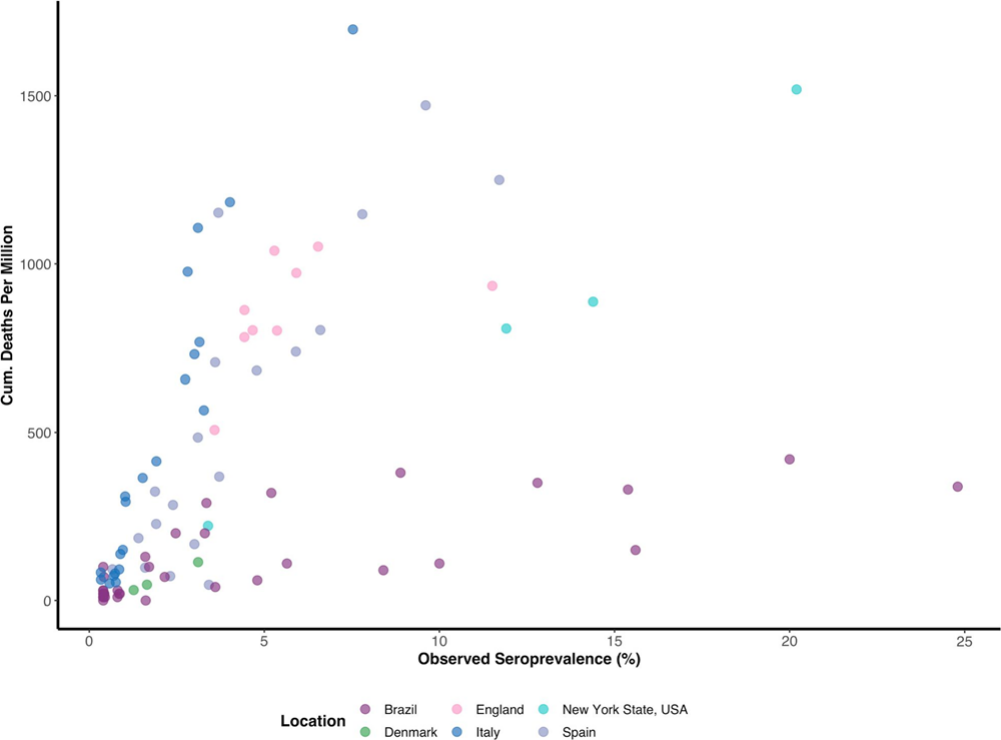
First-wave data: mortality versus seroprevalence. Relationship between seroprevalence and COVID-19 mortality per 1,000,000 among surveys which could be broken down by region.

We applied our model to 10 example serologic surveys conducted after the first wave. These were selected for being representative of the general population in a region or a country, and for availability of information on COVID-19 deaths in the area and the serologic test sensitivity and specificity (Supplementary Table 2). We did not include surveys after August 1st 2020, since dexamethasone and other changes in clinical practice occurred after this time^26^. These changes may have altered the IFR and our model assumes a constant IFR over time, The overall observed seroprevalence among the studies at the time ranged from ~1.6% in Zurich, Switzerland to 12% in New York State, USA, while the overall crude IFR ranged from 0.33% in Denmark to 2.3% in Italy (Table 1). Age seroprevalence did not follow a consistent pattern across settings: infection rates were relatively constant in some studies (e.g. Brazil) while increasing or decreasing with age in others (e.g. Spain and England, respectively; Supplementary Fig. 2). Seroprevalence was strongly correlated with cumulative mortality when data were stratified by regions within a serologic study (median correlation coefficient = 0.91, Fig. 6). However, the slope of the seroprevalence-mortality relationship varied considerably between studies (*p* < 0.001), suggesting differences in one or more of: the serologic test performance, deaths reporting, true IFR, or sampling bias (Fig. 6). Full model posterior estimates are given in Supplementary Tables 3, 4, 5, 6, Supplementary Figs. 3, 4, 5 and Supplementary Data 1.

**Table 1:** Overall infection fatality ratio estimates among the included studies.

	Data					Model estimates			
Study location	Cumulative COVID-19 deaths	Reported Seroprevalence (dates)	Serostudy Sensitivity (%) (T+/D+ or 95% CrI)	Serostudy Specificity (%) (T−/D− or 95% Crl)	Crude IFR (95 CI%)	Sensitivity (%) (95% CrI)	Specificity(%) (95% CrI)	IFR without Seroreversion (95% CrI)	IFR with Seroreversion^β^ (95% CrI)
Brazil*	51,179	2.42% (Jun. 04–Jun. 07)	85.14 (81.93, 87.97)	99.72 (99.55, 99.85)	0.99 (0.92, 1.06)	85.28 (82.12, 88.14)	99.76 (99.62, 99.87)	1.03 (0.93, 1.15)	0.99 (0.89, 1.12)
Denmark*	463	2.4% (Apr. 27–May 03)	82.09 (75.51, 87.58)	99.25 (98.94, 99.56)	0.33 (0.23, 0.48)	82.45 (76.11, 87.8)	99.16 (98.73, 99.46)	0.54 (0.38, 1.02)	0.51 (0.36, 0.97)
England*	48,301	5.94% (Jun. 20–Jul. 13)	78.4 (65.68, 88.15)	99.44 (99.11, 99.71)	1.42 (1.39, 1.46)	79.48 (68.74, 88.93)	99.59 (99.34, 99.78)	1.18 (1.02, 1.34)	1.07 (0.84, 1.24)
Italy*^, α^	34,610	2.44% (May 25–Jul. 15)	96.04 (89.84, 99.05)	99.7 (99.59, 99.79)	2.3 (1.94, 2.72)	96.42 (90.93, 99.13)	99.69 (99.57, 99.78)	2.53 (2.31, 2.78)	2.40 (2.18, 2.63)
Netherlands	5767	5.5% (May 10–May 20)	98.28 (171/174)	99.65 (281/282)	0.6 (0.58, 0.63)	98.23 (95.61, 99.52)	99.83 (99.43, 99.98)	0.62 (0.58, 0.69)	0.59 (0.55, 0.65)
Spain*	28,116	5.27% (Jun. 08–Jun. 22)	81.84 (75.67, 87.01)	98.79 (98.55, 99.02)	1.12 (1.08, 1.16)	84.72 (83.08, 88.36)	99.05 (98.86, 99.21)	1.14 (1.08, 1.22)	1.08 (1.01, 1.16)
Sweden	4992	7.1% (Jun. 08–Jun. 12)	99.36 (156/157)	98.89 (267/270)	0.68 (0.46, 1)	99.28 (97.23, 99.93)	99.17 (98.12, 99.77)	1.02 (0.87, 1.37)	0.98 (0.83, 1.35)
Geneva, Switzerland	262	10.84% (May 03–May 10)	91.16 (165/181)	100 (176/176)	0.48 (0.42, 0.56)	91.47 (87, 94.89)	99.89 (98.82, 100)	0.49 (0.42, 0.59)	0.47 (0.4, 0.57)
Zurich, Switzerland	124	1.59% (May 01–May 31)	90.74 (49/54)	99.89 (5,497/5,503)	0.51 (0.45, 0.58)	91.77 (83.39, 96.89)	99.87 (99.74, 99.95)	0.52 (0.41, 0.67)	0.50 (0.39, 0.64)
New York State, USA*	17,718	12.1% (Apr. 19–Apr. 28)	89.39 (85.57, 92.55)	98.73 (98.15, 99.27)	0.75 (0.74, 0.76)	89.66 (85.9, 92.68)	98.7 (98.05, 99.2)	0.78 (0.73, 0.84)	0.76 (0.7, 0.81)

The data columns (left) contain data and parameters used to calculate the crude IFR, the model columns (right) contain the posterior estimates from the full model. Citations for all the data sources are in Supplementary Table 2. The reported seroprevalences are listed along with the most recent dates for the seroprevalence survey. Cumulative deaths are summed to the mid-date of the most recent seroprevalence survey, and were usually confirmed COVID-19 test-positive patients except in England, which also reported probable COVID-19 deaths (individuals without test results but with COVID-19 on the death certificate). For the six studies with regional data, estimates of specificity and sensitivity were from analysis of regional data: posterior distributions with the median and 95% credible intervals are provided in place of the serologic test validation numbers (*). Model-estimated posterior sensitivity and specificity are indicated for the model with seroreversion, although these estimates were similar for both models (Supplementary Table 5). Overall IFR estimates were calculated by standardising the age-specific IFR estimates according to the inferred age-specific attack rate and the population demography with respect to the age groups used in the model (median, (95% Credible Intervals)). For comparison, the overall IFR estimates calculated by standardising for solely the demography and assuming the same attack rate in each age group are provided in Supplementary Table 3.

Serologic test performance is measured by the sensitivity and specificity (*T*+ test positive, *D*+ true positives, *T*− test negative, *D*− true negatives).

^α^Serovalidation data for the Italian serosurvey using the Abbott assay were not validated within the same study; here we used an alternative study testing the same assay.

^β^Assuming an extreme rate of seroreversion for sensitivity analysis based on the Abbott assay. The true seroreversion rates in these studies are unknown, but are likely less extreme, particularly if the Abbott assay was not used (only the Italy study used the Abbott assay.

### First-wave IFR estimates

We first re-estimated the specificity of the serologic assay for each study with regional data (Brazil, England, Denmark, Italy, Spain, and New York State), based on the relationship between seroprevalence and mortality (Supplementary Fig. 7). We found that the estimated specificity often differed from the reported values (Supplementary Table 5). For example, the study in Spain reported 100% specificity (95% CI: 97.7–100%) but our estimated value was 98.79% (95% CI: 98.55–99.02%; Table 1; Supplementary Fig. 6). We used our updated test performance estimates as informative priors in the model-based analysis of the IFR in each survey.

Our statistical IFR model found that 2/10 included studies (Denmark; Sweden) had highly uncertain IFRs. These results were due to low sensitivity or specificity of the serologic tests, leading to a large number of false positives or false negatives relative to the observed seroprevalence (Table 1). The overall IFR ranged from 0.49 to 2.53% (Table 1). In a subset of surveys (Switzerland, Netherlands, Spain, and New York), the crude IFRs closely matched the modelled IFRs, consistent with serologic studies being conducted after the first wave. For Italy, where the survey was conducted several months after the peak of the epidemic, including seroreversion had some effect on the estimated IFR (declining from 2.53 to 2.40%). Seroreversion had relatively little effect on IFR estimates for other studies, despite the assumption of rapid seroreversion in this sensitivity analysis, which was likely to be faster than the true value in most studies.

### Age-stratified IFR estimates

**Fig. 7:**
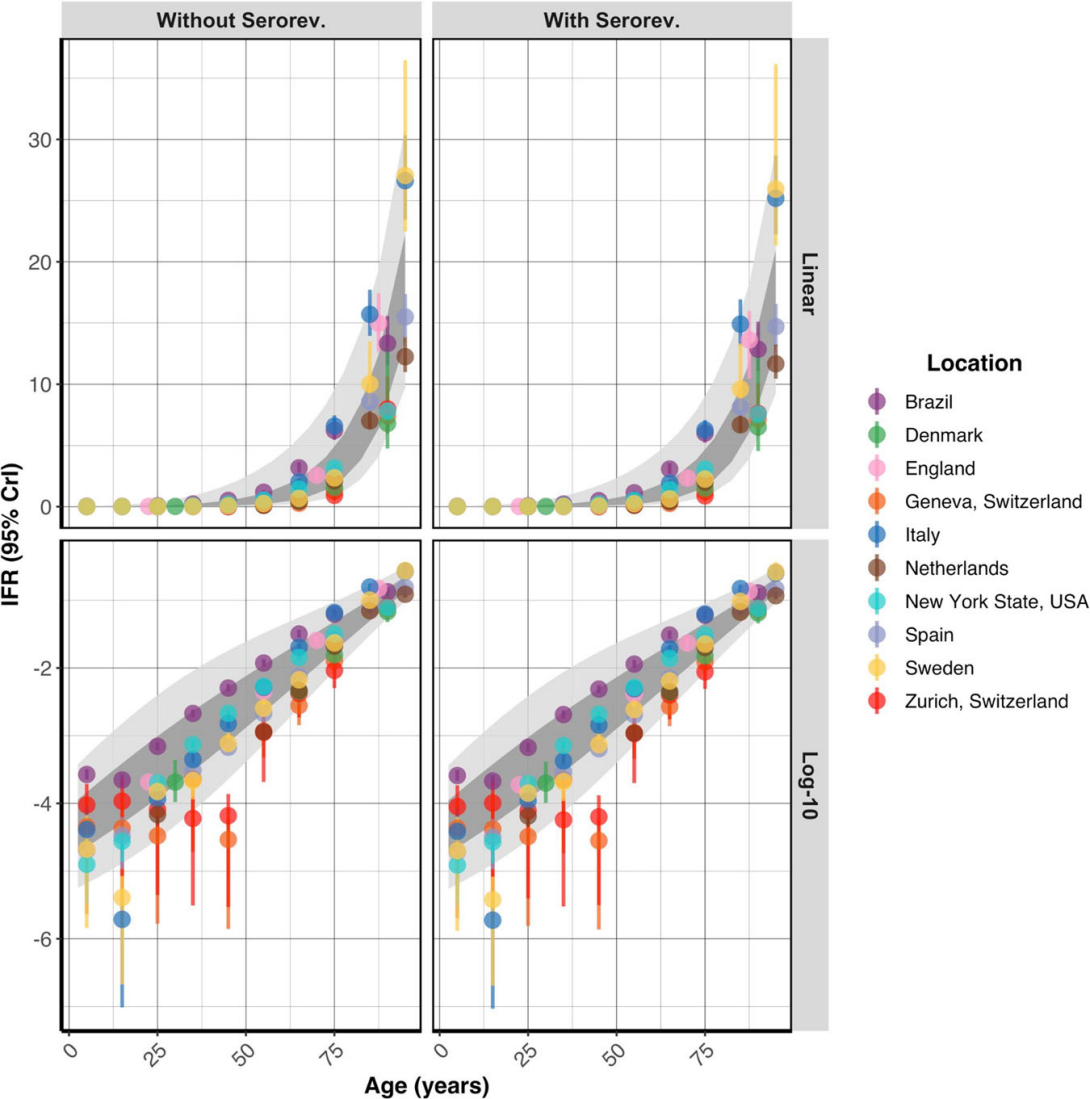
Age-stratified infection fatality ratio estimate. The age-specific modelled IFR (%) median and 95% credible interval estimates with and without seroreversion are plotted on a linear and log-10 scale (mean age within each age group plotted). The 95% prediction intervals (light grey) and the 80% prediction intervals (dark grey) calculated from the age-specific pooled-IFR estimates are shown for each model. The IFR increases in a log-linear fashion with age.

**Fig. 8:**
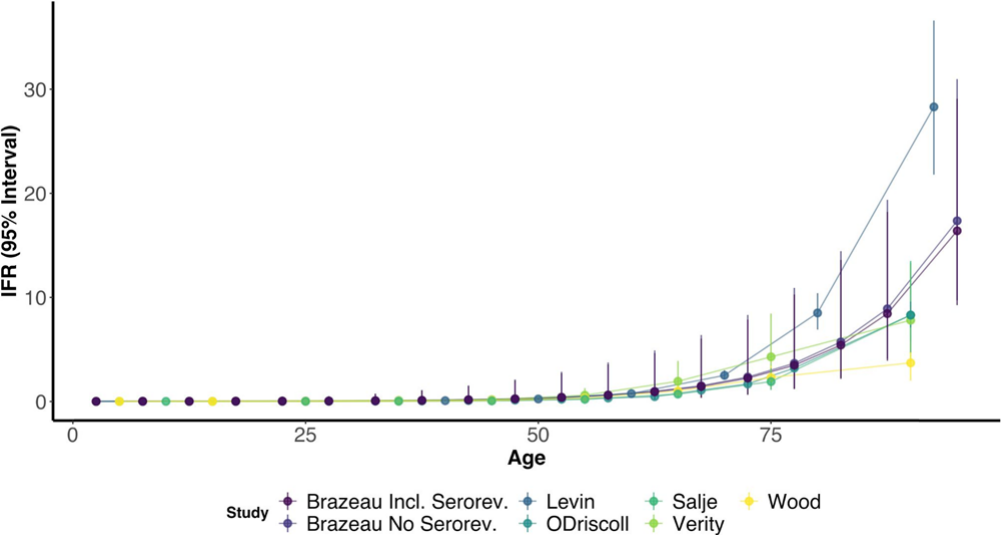
Comparison of age-specific COVID-19 IFR estimates during the first-wave. We compare estimates from the current study (Brazeau; including seroversion (Incl. Serorev.) vs. excluding seroreversion (No Serorev.)) with and without seroreversion, Levin et al.^10^, Salje et al.^6^, Wood et al.^5^, O’Driscoll et al.^2^ and Verity et al.^4^. Of note, studies used different statistical (i.e. frequentist versus bayesian) and methodological approaches that make the 95% confidence or credible intervals not directly comparable.

We calculated a pooled IFR estimate for 5-year age bands and predictive intervals, showing the plausible range of IFRs that can be expected in a new study population (Table 2, Supplementary Fig. 8). Analysis of first-wave data showed that IFRs increased steeply with age, following an approximately log-linear relationship (Fig. 7) with IFR in 5–9 year olds being around 0.01%, increasing to close to 1% in 60–65 year olds and >15% in over 90 year olds (estimates not allowing for seroreversion). We standardised these age-specific IFR estimates across four age-demographics representative of countries in the LIC, LMIC, UMIC, and HIC wealth brackets, demonstrating that the IFR is expected to range from 0.24% in an average LIC to 1.1% in a HIC due to the age structure in the population (Table 2; Supplementary Note 4). We also contrasted our estimates to previous estimates of the IFR (Fig. 8).

**Table 2:** Pooled estimates of the infection fatality ratio.

Age-band (years)	IFR (%) without seroreversion (95% PI)	IFR (%) with seroreversion (95% PI)
0–4	0 (0, 0.04)	0 (0, 0.04)
5–9	0.01 (0, 0.07)	0.01 (0, 0.07)
10–14	0.01 (0, 0.12)	0.01 (0, 0.11)
15–19	0.02 (0, 0.2)	0.02 (0, 0.19)
20–24	0.03 (0, 0.32)	0.03 (0, 0.31)
25–29	0.04 (0, 0.5)	0.04 (0, 0.48)
30–34	0.07 (0.01, 0.75)	0.06 (0.01, 0.72)
35–39	0.1 (0.01, 1.09)	0.1 (0.01, 1.05)
40–44	0.16 (0.02, 1.54)	0.16 (0.02, 1.47)
45–49	0.25 (0.03, 2.11)	0.24 (0.03, 2.02)
50–54	0.4 (0.06, 2.84)	0.38 (0.05, 2.7)
55–59	0.62 (0.1, 3.75)	0.59 (0.1, 3.56)
60–64	0.96 (0.19, 4.9)	0.92 (0.18, 4.64)
65–69	1.5 (0.35, 6.38)	1.43 (0.34, 6.03)
70–74	2.34 (0.66, 8.31)	2.23 (0.63, 7.85)
75–79	3.66 (1.23, 10.9)	3.47 (1.18, 10.27)
80–84	5.71 (2.26, 14.44)	5.41 (2.16, 13.59)
85–89	8.9 (4.09, 19.37)	8.43 (3.91, 18.21)
90+	17.36 (9.73, 30.97)	16.4 (9.25, 29.08)
**Overall (LIC)**	*0.24 (0.15, 0.43)*	*0.23 (0.14, 0.41)*
**Overall (LMIC)**	*0.4 (0.27, 0.68)*	*0.39 (0.25, 0.65)*
**Overall (UMIC)**	*0.62 (0.41, 1.01)*	*0.59 (0.39, 0.97)*
**Overall (HIC)**	*1.16 (0.79, 1.82)*	*1.1 (0.75, 1.72)*

Bold and Italic values represent the overall numbers at the end.

IFR estimates were calculated by combining study- and age-specific IFR estimates in a log-linear model. The median predicted estimate and corresponding 95% prediction intervals (PIs) are shown above. Predictive intervals were used to express the plausible range of IFRs that can be expected in a new study population, rather than showing our degree of certainty of our estimates with confidence intervals. For the 90+ age group, we assumed a maximum age of 100 years. The overall IFR estimates were standardised by the population structure in a representative low-income country (LIC), low-middle income country (LMIC), upper-middle income country (UMIC), and high-income country (HIC), assuming equal attack rates across age groups.

## Discussion

Estimating the IFR of a novel infectious disease is inherently challenging due to the dynamic and imperfect nature of the available data. Here we have developed a statistical framework to account for key uncertainties in the data to provide robust estimates of the IFR of COVID-19. We found that a model-based approach was needed in order to account for biases in estimating the IFR even after adjusting for test sensitivity and specificity. For example, we found that the IFR was typically biased downwards for serosurveys conducted early in the epidemic, when infections are growing, whilst the IFR was typically biased upwards when serosurveys were conducted after the initial epidemic wave passed and seroreversion became more likely (i.e. decay in antibody titres leading initially seropositive individuals to become seronegative). As an epidemic progresses, seroreversion leads to an increasing loss of sensitivity to detect previously infected individuals using serologic surveys^27,28^. However, where data are available on the time to seroreversion among previously infected individuals, our statistical framework is able to recover the correct IFR.

Our model showed that it propagated uncertainty as our estimates of age-specific and overall IFRs were more uncertain among first-wave studies reporting both a low seroprevalence and low specificity. This increased level of uncertainty is appropriate, as sensitivity and specificity can skew estimates of the cumulative infection incidence derived from seroprevalence surveys, particularly when infection is not widespread and positive results may be dominated by false positives. For example, Denmark appears to have a lower IFR than other countries from crude IFR estimates (0.33%) but was consistent with other countries after we re-estimated test specificity from regional data and incorporated uncertainty: 0.54% (0.38, 1.02). However, a limitation of our model is that we did not explicitly account for death as a competing hazard with seroconversion, as the observed seroprevalence (i.e. model data input) is inherently calculated among surviving individuals. As a result, if observed seroprevalences are artificially low due to survivor bias, we may overestimate the IFR in specific groups. Resolving this competing hazard likely requires individual-level data but may benefit future IFR statistical models.

Applying our model to high-quality studies from the first-wave of the COVID-19 pandemic, we found a comparatively consistent pattern across ages, with age-stratified IFRs demonstrating an approximately log-linear relationship with increasing age. These results are consistent both with early reports^4–6^ and more recent meta-analyses^2,10^, although our pooled estimate of the IFR in high-income countries is slightly higher. Applying these estimates directly to a specific country should be done cautiously, as factors other than age (e.g. healthcare capacity, intervention uptake, etc.) will affect the IFR. In addition, our selected studies do not include representation from LMICs, which further limits generalisability. However, comparing IFR estimates amongst different demographies does capture and explain some of the observed IFR global variation.

Importantly, our model demonstrated that first-wave estimates of the IFR were relatively similar when seroreversion was and was not accounted for in the analysis. Our seroreversion parameters were estimated using data on serial antibody titres with the Abbott N-antibody assay from previously diagnosed non-hospitalised COVID-19 patients^25^. These parameter estimates likely represent the maximum effect of seroreversion given that non-hospitalised individuals tend to serorevert faster than their severe disease counterparts^29^, the Abbott assay has known decreases in sensitivity over time, and our model assumption that everyone will serorevert. Within this framework, we estimated that over 6 months, an average of 48.25% individuals would serorevert after seroconverting when tested with the Abbott assay. This loss of sensitivity over 6 months exceeds that of a recent estimate of 33% for the Abbott N-antibody assay, which may be due to differences in disease severity and age between the study populations^30^. Despite this deliberately pessimistic approach the IFR was only marginally decreased when considering seroreversion, indicating that not enough time had passed for a substantial proportion of infected people to serorevert. This suggests that first-wave estimates of the IFR were not biased despite not explicitly encoding this observed phenomenon into their models^2,4,10^. Our model was able to accurately infer IFR from many epidemic shapes, and only inferred IFR less accurately when there was exponential growth and fewer deaths in younger age groups. This limitation is largely mitigated by the consideration that none of the included first-wave studies had uncontrolled exponential growth over the first wave.

Accounting for seroreversion is becoming increasingly important as time passes since the first epidemic waves of SARS-CoV-2. To estimate IFR up to the current day, our model needs further development to incorporate potential change in IFR over time. For example in the UK, IFR was approximately halved by the end of 2020 compared to the first wave^31^, likely due to improvements in treatment and clinical practice. IFR could potentially also increase, for example when health systems become overwhelmed. Time-varying IFR estimates require repeated serosurveys over time in the same population.

Models can only perform as well as their inputted data, and there are limitations in the serologic and death data we used from the first wave. We made the deliberate decision to focus on seroprevalence studies that met a high bar for inclusion to limit the amount of sampling bias introduced into our overall IFR calculations compared to other studies^32^. For example, we excluded samples of patients attending clinical settings during the pandemic, judging that they were more likely to be exposed to the virus. This may explain why our estimates of IFR are slightly higher than another meta-analysis^2^. Despite this high threshold, several included studies were not ideal due to numerous factors: 4/10 (Denmark; Netherlands; Sweden; Zurich, Switzerland) had seroprevalence data from blood donors, which may not be representative of the general population. In addition, the seroprevalence study in New York State recruited participants at grocery stores, which may represent a biased study population (Supplementary Table 2). A recent study estimated a higher IFR in New York City based on case data and assuming the seroprevalence in shoppers was higher than the general population^33^. Similarly, quantifying deaths from COVID-19 has been challenging for many countries, due to death counts being revised over time or countries differing in approaches to counting COVID-19 deaths. In some instances, particularly in LICs and LMICs, deaths have been underreported^34,35^. Large increases in excess deaths have also been noted during COVID-19 epidemic waves, suggesting under-diagnosis^36–38^. Most countries included in our study reported deaths amongst test-positive cases only, but England also reported probable COVID-19 deaths^39^. In England, most probable deaths without laboratory confirmation occurred in care homes, so the difference between using probable and confirmed deaths is likely to be greater for IFR estimates including care-home deaths. Separately, an analysis found a positive relationship between COVID-19 mortality rates and excess deaths mortality rates in high-income countries, suggesting that missed COVID-19 deaths did not explain differences in the mortality rates between these countries^2^. Inclusion of non-representative data can lead to biased fits given the limitations of seroprevalence study designs during the first-wave^19,40^. Including a larger number of studies does not make a better meta-analysis, and future studies should carefully assess potential bias caused by seroprevalence sampling strategies or regions with known death underreporting.

We showed that information on the specificity of a seroassay in the general population could be determined by contrasting trends of cumulative deaths versus seroprevalence across regions. Identifying the specificity of a seroassay in the general population is critical particularly when seroprevalence is low and may consist of more false than true positives. In addition, we found that our model was sensitive to the prior placed on specificity. Among the studies that had region-disaggregated seroprevalence data, our estimates of test specificity were sometimes different from estimates derived from assay validation studies. Collectively, this suggests that the large heterogeneities in the IFR between populations may in part be due to differences in serologic assay performance. Serologic test sensitivity may also be lower in the general population than in assay validation studies, as assays are often validated in hospitalised patients with more recent severe disease, whilst the majority of infections in the general population have milder disease and may produce a lower^41^ or sometimes no antibody response^42^. Additionally, assay sensitivity may be lower in the general population due to cross-reactive antibodies and/or a cell-mediated immune response^43,44^.

In summary, we provide a statistical framework to estimate COVID-19 IFR that accounts for seroreversion as part of the IFR estimation as well as simultaneously accounting for uncertainty in serologic test characteristics, variation in age-specific attack rates, and delays from infection to death and seroconversion. We additionally show the possibility of estimating test specificity from regional data breakdowns. We estimate that the overall COVID-19 IFR ranges from 0.15–0.43% in low-income countries to 0.79–1.82% in high-income countries, with the differences in those ranges reflecting the older demography of high-income settings. The IFR is also likely to vary depending on available healthcare and underlying health conditions. Our results suggest that the overall risk of death from COVID-19 doubles with approximately every 8 years of age. Our estimates of the IFR of COVID-19 are consistent with early estimates and remain substantially higher than IFR estimates for seasonal influenza (<0.1% in the USA)^45^. As the pandemic wanes and vaccines are allocated, it is important to update previous IFR estimates from the first-wave of the pandemic to reflect on and justify past interventions and risk mitigation analyses^23,46^.

## Supplementary information

Supplementary Information

Supplementary Data 1

Description of Additional Supplementary Files

Reporting Summary

## Data Availability

All of the data, including source data for the figures and tables, are publicly available on Github (https://github.com/mrc-ide/reestimate_covidIFR_analysis)^22^, and original sources are provided in Supplementary Table 2, with the exception of raw data for the onset to seroreversion analysis. The onset to seroreversion data cannot be made public as it contains individual-level and identifiable patient data and is available on request (S.J.).

## Code availability

The IFR statistical model, and accompanying code, is available as a standalone R-package at Github: ‘mrc-ide/COVIDCurve’ (v0.5.0)^22^.
