# Supplementary material for: Estimating the COVID-19 infection fatality ratio accounting for seroreversion using statistical modelling
Source: Commun Med (Lond). 2022 May 19;2:54. doi: 10.1038/s43856-022-00106-7 (PMC9120146; doi:10.1038/s43856-022-00106-7)
Supplement: Supplementary file 1 — Supplementary Information [file 43856_2022_106_MOESM1_ESM.pdf]

Supplementary Information

Nicholas F Brazeau, Robert Verity, Sara Jenks, Han Fu, Charles Whittaker, Peter Winskill, Ilaria Dorigatti, Patrick Walker, Steven Riley, Ricardo Schnekenberg, Henrique Hoeltgebaum, Thomas A Mellan, Swapnil Mishra, H Juliette T Unwin, Oliver J Watson, Zulma M Cucunubá, Marc Baguelin, Lilith Whittles, Samir Bhatt, Azra C Ghani, Neil M Ferguson, Lucy C Okell.

SUPPLEMENTARY MATERIALS

Supplementary Methods 2

Symptom onset to seroreversion parameter estimation 2

Onset-outcome delay distributions and serological test characteristics 2

Obtaining specificity priors from regional data 3

Supplementary Note 1. Comparison of the number of serosurveys 5

Supplementary Note 2. First wave data 6

Seroprevalence data 6

COVID-19 mortality data and demographics 6

Supplementary Note 3. IFR statistical model posterior estimates 9

Posterior parameter estimates 9

Posterior predictive checks 12

Specificity estimates from regional data 16

Supplementary Note 4. Pooled IFR calculation 18

Supplementary References 20

Supplementary Methods

Symptom onset to seroreversion parameter estimation

We estimated the time to seroreversion using an extended dataset from the previously published longitudinal Abbott SARS-CoV-2 IgG assay data[1] as described in the main text. Of the 109 convalescent participants analyzed, eight did not seroconvert during follow-up (*i.e.* they were negative at baseline and remained so during the follow-up period). Among the final 101 participants, 72 were female and the median age was 47 years (range: 21 - 65 years). Seronegative status (*i.e.* the outcome) was defined as titres below an optical density of 1.4.[1] From the Weibull survival model estimated using the `survival` R-package, [2,3] we found that the Weibull shape and scale parameters were 2.32 and 215.5., respectively. In order to conform with the mathematical derivation of the model (**Equation 16** above), we subtracted 13.3 (days) from the Weibull scale parameter to account for the time of symptom onset to seroconversion (as an approximation).[4]

Onset-outcome delay distributions and serological test characteristics

Prior distributions are given in Supplementary Table 1. The delay from onset of symptoms to death was assumed to follow a gamma distribution with a mean of 14.8 days and coefficient of variation of 0.85.[5] The delay from onset of symptoms to seroconversion was assumed to follow an exponential distribution with a mean time of seroconversion of 13.3 days.[6] For both distributions, the incubation period was assumed to be fixed at five days,[7,8] thereby providing overall means of onset of infection to death and seroconversion of 19.8 and 18.3 days, respectively. Priors for the onset-delay means were truncated Gaussian distributions while the onset-to-death coefficient of variation had a Beta distributed prior (Supplementary Table 1). When seroreversion was considered, the prior for the rate of seroreversion after seroconversion was assumed to have a truncated Gaussian distribution.

The prior distributions for the age-specific IFR estimates were assumed to be uniform with minimum and maximum values of 0% and 40%, respectively. The 40% maximum was selected as not more than three-times the upper credible interval of three early IFR studies.[9–11]

Spline curves were fit with five knots to estimate the incidence of infections over time, with uniform priors on the x- and y-axis positions. The minimum and maximum values of the y-axis positions were zero and the total population size, respectively, while the maximum x-axis position was allowed to range from fourteen days prior to the last observed death date to the last observed death date.

To estimate the proportion of infections occurring in each age group, we assumed that the attack rate was proportional to the population fraction within that age group multiplied by an additional scaling parameter assumed to follow a truncated Gaussian distribution. The modelled seroprevalence by age was fit to the age specific seroprevalence data. Given that serostudies were conducted over a given timeframe (*e.g.* two-weeks), we took the average of the model seroprevalences over the serostudy time-period to propagate uncertainty in collection times. These modelled prevalences were then compared with the reported seroprevalence.

The prior distributions for the sensitivity and specificity were assumed to follow beta distributions based on the number of true positives that were correctly identified, the number of true negatives correctly identified, and the total number of samples tested. To allow for some uncertainty -- even among studies that found 100% specificity during serovalidation -- we added 0.5 to our Beta scale and shape parameters (the Jeffreys prior). For the subset of studies where regional seroprevalence information was available (6/10), we used the posterior estimates of sensitivity and specificity from the region-based models (see below) to re-inform the priors for the IFR model. The new beta distribution shape parameters were calculated from the regional-based model sensitivity and specificity posteriors using a method of moments approach with `fitdistplusr` R-package [12] (**Supplementary table 5**).

During model fitting, 10,000 burnin-in and 10,000 sampling iterations with 50 rungs across 10 chains were considered. The thermodynamic power between rungs was set to 3.0 to ensure high swap rates among the hotter chains for all models except: Brazil, England, Spain, Italy, New York State no-seroreversion and seroreversion models and Brazil, England, Italy, Sweden and New York State care-homes excluded models where the thermodynamic power was set to 2.0.

Obtaining specificity priors from regional data

A simplified version of the main statistical model described above was used to estimate serological test specificity in studies where data were available by region within the larger serosurvey. As before, we incorporated test sensitivity and specificity into this model using the reported validation data from each study as priors, and we then estimated regional and age-specific seroprevalence and deaths. Specificity and sensitivity were assumed constant across regions and ages. The age-specific IFRs were assumed to be the same in each region, but incorporated region-specific demography so that the overall regional IFRs could vary (*e.g.* London, England has a much younger population than the rest of the country, and therefore a lower overall IFR). The delays from onset-death and onset-seroconversion were removed for analytical tractability, and we used cumulative mortality data up to the midpoint of the serosurvey, making the assumption that most deaths and seroconversion had occurred by the time of the serosurvey. This assumption is reasonable for serosurveys conducted some weeks after the first wave of the epidemic. We did not have seroprevalence data broken down by both age and region at the same time, but we fit the model to both the marginal age and regional seroprevalence, assuming the same risk ratios of being seropositive by age in all regions. We did not include seroreversion in this analysis. The model was fit with RStan using 4 chains, each having 10,000 burn-in and 10,000 sampling iterations [13]. The underlying code is available on Github (mrc-ide/reestimate_covidIFR_analysis/analyses/Rgn_Mod_Stan). Code is also available in the same folder showing simulation of an epidemic with different regional attack rates, in order to test model performance (Figures 4B & 4C).

Parameter	Count	Distribution
Age-Specific IFRs	Number of Age Groups	Uniform(0,1); Uniform(0, 0.4)
Knots	5	Uniform(0,1); Uniform(E-14, E)
Spline Y-Positions	5	Uniform(0,1); Uniform(0, popN)
Attack Rate By Age	Number of Age Groups	Truncated-Normal(1, 0.05)<br>Bounds: 0.5, 1.5
Mean of Onset from Infection to Seroconversion	1	Truncated-Normal(18.3, 0.1)<br>Bounds: 16, 21
Mean of Onset from Infection to Death	1	Truncated-Normal(19.8, 0.1)<br>Bounds: 18, 20
Coefficient of Variation of Onset from Infection to Death	1	Beta(2550, 450)
Weibull Shape Pattern for Seroreversion	1	Truncated-Normal(2.32, 0.5)<br>Bounds: 2, 5
Weibull Scale Pattern for Seroreversionn	1	Truncated-Normal(202.2, 0.1)<br>Bounds: 127, 133

Supplementary Table 1 - Prior distributions used in the IFR statistical model: For each parameter, the prior distribution is indicated alongside the parameter count. For the age-specific infection fatality ratios (IFRs), younger age group IFRs were reparameterized as risk relative to the oldest age group in order to improve model mixing. Similarly, the spline knots were reparameterized relative to the last observed day, where *E* was the last observed day and *E-14* was fourteen days prior. The first knot was always fixed at one to allow for a common time origin. The spline y-positions were similarly set relative to the third y-position, which was allowed to range between zero and the approximate size of the population (popN).

Supplementary Note 1. Comparison of the number of serosurveys

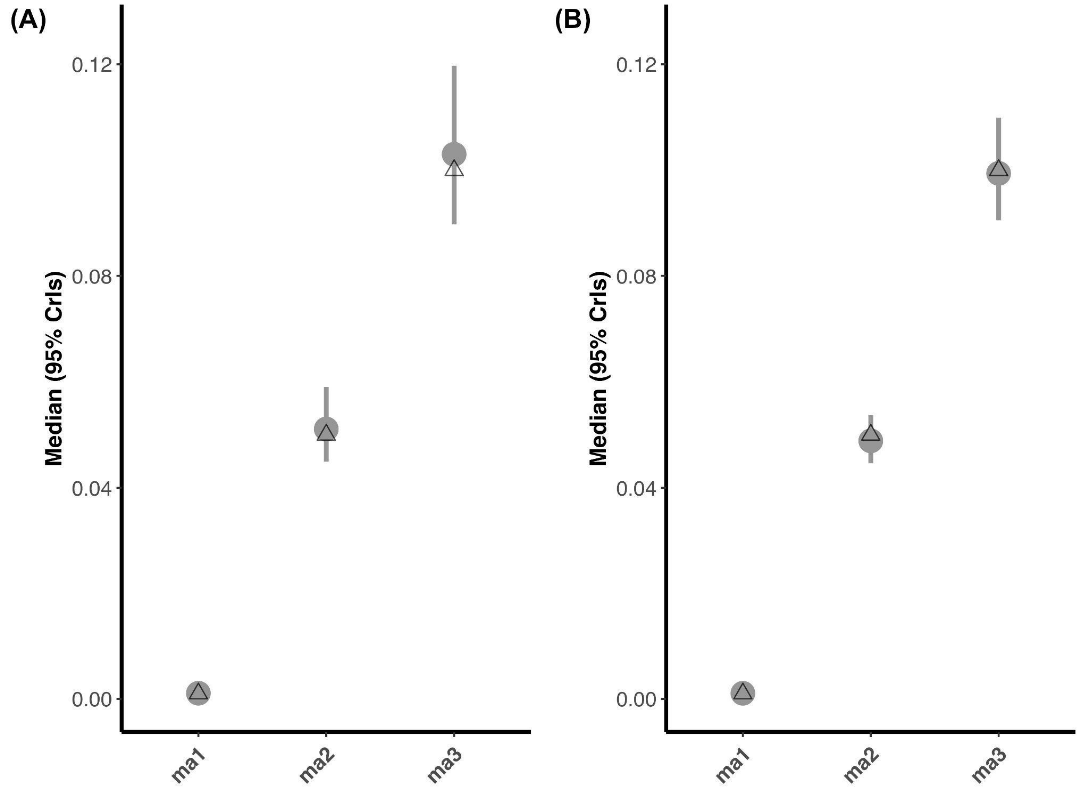

Supplementary Figure 1 - Estimating IFR from one versus two serosurveys. Data was simulated for three age groups (ma1, ma2, ma3) with IFRs of 0.1%, 1%, and 10%, respectively. Follow-up time was assumed to be 200 days. The simulations differed in the number of simulated serosureys with (**A**) consisting of a single serosurvey ranging from days 145-155 versus (**B**) consisting of two serosurveys: days 120-130 and 170-180, respectively. In both simulations, we assume that 0.1% of the population was sampled and that the simulated serologic test had a sensitivity and specificity of 0.85% and 0.95%, respectively. Although the statistical model can capture the true simulated IFR values in the 95% credible intervals (grey) in both scenarios, there is greater uncertainty when only a single serosurvey is considered. The IFR values are shown as a probability.

Supplementary Note 2. First wave data

Seroprevalence data

Identification of relevant serological studies was carried out using the `SeroTracker` dashboard [14], a continuously updated systematic review. Observed seroprevalences, disaggregated by age and location where possible, were extracted. For the majority of studies (7/10), raw counts for the number of seropositives and number tested were available. However, this information was not available for three studies (conducted in Italy, Sweden and Denmark) and reported seroprevalence was extracted instead, alongside the stated confidence intervals. As a result, we used the reported confidence intervals to capture standard errors and used a logit-transformation instead of a binomial approach in our statistical model likelihood (*i.e.* a normal likelihood on the logit-scale). Further uncertainties in the data extraction process include:

- For the Swedish study, seroprevalence data was extracted from public health reports that were being constantly updated and did not provide exact dates of the period in which the serostudy was conducted. It was therefore assumed that the serostudy was carried out in contiguous weeks across the study period, based on the provided figure in the report (accessed September 11, 2020).
- In 5/10 studies (Zurich, Switzerland; Denmark; Sweden; Netherlands [second time point]), the age-specific seroprevalences and sample sizes were from broad age groups or did not overlap well with reported death age groups, or only a national prevalence was available. As a result, we assumed a constant attack rate (seroprevalence) across age groups for these studies.
- In instances where the reported serologic age groups did not directly match with reported cumulative death age groups, we assumed similar seroprevalence values in contiguous age groups or used the overall average for missing age groups.

COVID-19 mortality data and demographics

In a limited number of cases, Ministries of Health have retrospectively removed previously reported deaths, leading to negative reported deaths on particular days - these observations were set to zero. On May 18 for the New York data, nearly 4,500 deaths were retrospectively added, which caused an outlier in daily observed deaths. Inclusion (not shown) or exclusion of this day's data resulted in very similar New York IFR estimates because it was difficult for the model to capture this spike. MCMC chains appeared much more stable when this day's data was excluded, so were used for the final result.

Demographic data was obtained in order to generate our standardized pooled IFR calculations for settings representative of each of the World Bank Income Strata - specifically Low Income Countries (LICs), Lower-Middle Income Countries (LMICs), Upper Middle Income Countries (UMICs) and High Income Countries (HICs). Consistent with previous analyses that have identified the representative countries in each of these strata [15], we selected Madagascar, Nicaragua, Grenada and Malta: information on their demographic structure was downloaded from the United Nations World Population Prospects 2019 site [16].

Study Location	Seroprevalence & Serovalidation Data	Number of Serostudy Timepoints	Age-Specific Death Data	Time-Series Death Data	Demographic Data
Brazil	Suppl Table 3 in [17], Laboratory methods section[18]	1	[19]	[19]	[20], CP Population Projections by Sex and Age
Denmark	Tables 1 & 3 in [21,22]	4	Age mortality table [23]	[24]	[25]
England	[26,27]	1	Table 2 [28]	[29]	[30]
Italy[+]	Table 1, Seroprevalence by Region; Table 2 Seroprevalnce by age[31,32].	1	[31,33]	[34]	[35]
Netherlands	Table 1, Seroprevalence by Age and Serovalidation in main text [36] ; Seroprevalence estimates in main text [37]	2	[38]	[39]	[40]
Spain	Serovalidation and Seroprevalence reports available via site [41,42]	3	[43]	[43]	[44]
Sweden	[45,46*]	8	[47]	[47]	[48]
Geneva, Switzerland	Table 1, ELISA IgG No. (%) GE cut-offs [49,50]	5	[51]	[51]	[52]
Zurich, Switzerland	Section: Prevalence of anti-SARS2 antibodies in prepandemic samples, Fig S2A, B; Section on Clinical and demographic characteristics of serologically confirmed SARS2 infected hospital patients and healthy donors, Table S1. [53]	2	[51]	[51]	[52]
New York State, USA	Table 1, Appendix part 1&2 [54]	1	[55]	[56,57]	Annual County and Resident Population Estimates by Selected Age Groups and Sex: April 1, 2010 to July 1, 2019 (CC-EST2019-AGESEX)[58]

Supplementary Table 2 - Data Extraction Sources: The sources of data for IFR estimation in the first wave (reference numbers). All data are publicly available. For some regions, population demographic data was taken from the City Population (CP) website: http://citypopulation.de/

* Sweden seroprevalences were limited April 20 - June 12, 2020 when multiple regions were considered (Jämtland-Härjedalen, Jönköping, Kalmar, Skåne, Stockholm, Uppsala, Västerbotten, Västra Götaland, and Örebro). These provinces include the majority of the Swedish population and were assumed to be nationally representative.

[+] Serovalidation data has not yet been released for the Italy study. As a result, serovalidation data from a comparable study in San Francisco, USA, which used the same Abbot seroassay, were used as priors for the regional model and re-estimation of the test sensitivity and specificity.

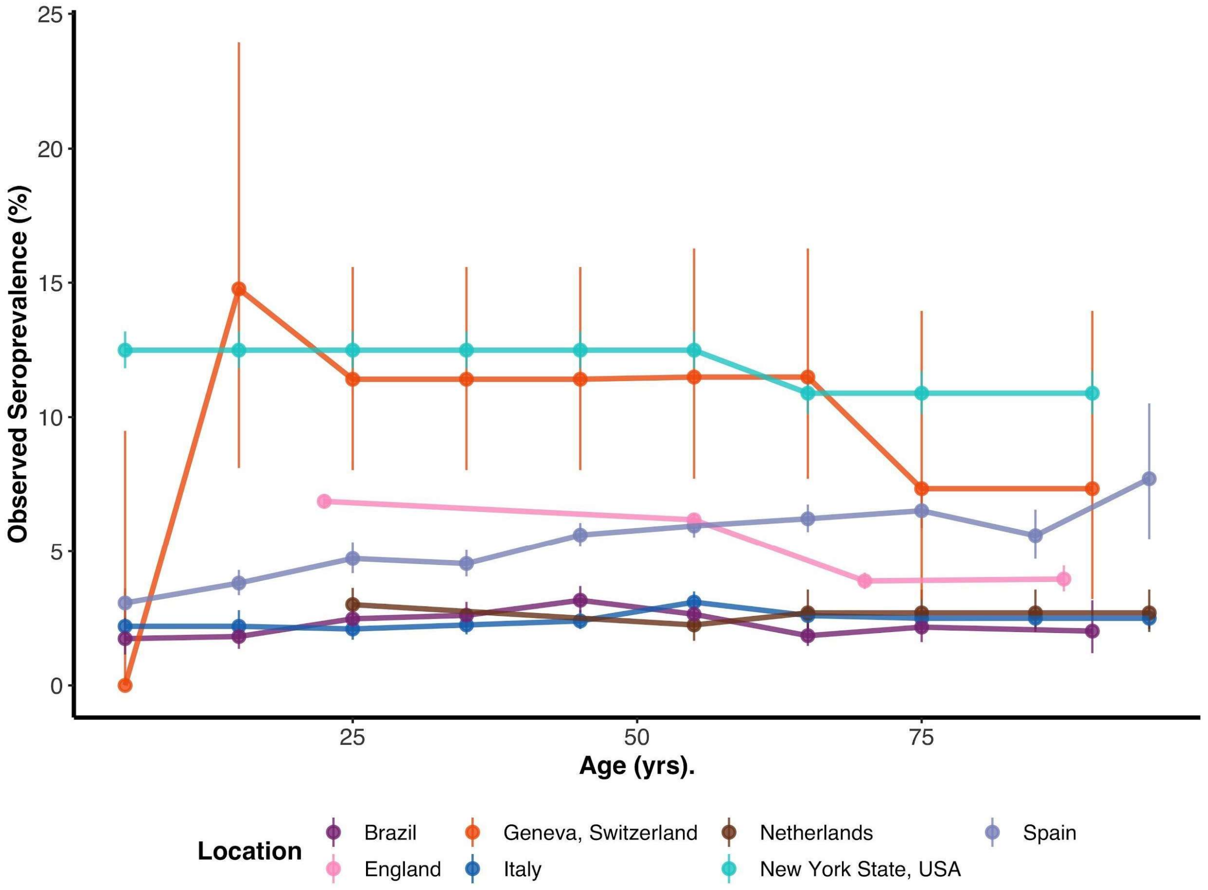

Supplementary Figure 2 - Seroprevalence versus Age: Observed seroprevalence by age (mean age within each age group plotted) and 95% confidence intervals for the seven studies where age-specific information was reported. The results of the latest seroprevalence survey are plotted among locations where multiple seroprevalence surveys had been conducted (except for the Netherlands where age specific seroprevalence was only available in the first survey). In our analysis we assumed a uniform seroprevalence across age groups among the remaining three studies included that did not contain age-specific seroprevalence data.

Supplementary Note 3. IFR statistical model posterior estimates

Posterior parameter estimates

Study Location	Crude IFR (95 CI%)	Pop.-Attack Rate Standardized Modelled IFR without Seroreversion (95% CrI)	Pop.-Attack Rate Standardized Modelled IFR with Seroreversion (95% CrI)	Pop. Standardized Modelled IFR without Seroreversion (95% CrI)	Pop. Standardized Modelled IFR with Seroreversion (95% CrI)
Brazil	0.99 (0.92, 1.06)	1.03 (0.93, 1.15)	0.99 (0.89, 1.12)	1.05 (0.95, 1.18)	1.01 (0.91, 1.15)
Denmark	0.33 (0.23, 0.48)	0.54 (0.38, 1.02)	0.51 (0.36, 0.97)	0.54 (0.38, 1.02)	0.52 (0.37, 0.96)
England	1.42 (1.39, 1.46)	1.18 (1.02, 1.34)	1.07 (0.84, 1.24)	1.52 (1.3, 1.75)	1.38 (1.07, 1.62)
Italy	2.3 (1.94, 2.72)	2.53 (2.31, 2.78)	2.4 (2.18, 2.63)	2.53 (2.29, 2.79)	2.4 (2.17, 2.65)
Netherlands	0.6 (0.58, 0.63)	0.62 (0.58, 0.69)	0.59 (0.55, 0.65)	0.62 (0.58, 0.68)	0.59 (0.55, 0.65)
Spain	1.12 (1.08, 1.16)	1.14 (1.08, 1.22)	1.08 (1.01, 1.16)	1.04 (0.97, 1.12)	0.99 (0.92, 1.07)
Sweden	0.68 (0.46, 1)	1.02 (0.87, 1.37)	0.98 (0.83, 1.35)	1.03 (0.87, 1.37)	0.98 (0.83, 1.36)
Geneva, Switzerland	0.48 (0.42, 0.56)	0.49 (0.42, 0.59)	0.47 (0.4, 0.57)	0.52 (0.43, 0.62)	0.5 (0.41, 0.61)
Zurich, Switzerland	0.51 (0.45, 0.58)	0.52 (0.41, 0.67)	0.5 (0.39, 0.64)	0.52 (0.41, 0.68)	0.5 (0.39, 0.64)
New York State, USA	0.75 (0.74, 0.76)	0.78 (0.73, 0.84)	0.76 (0.7, 0.81)	0.83 (0.78, 0.9)	0.81 (0.75, 0.88)

Supplementary Table 3 - Overall IFR Estimates when Standardized by Population Demography and Inferred Attack Rates: The overall IFR estimates were calculated by standardizing for demography and the inferred attack rate, following the definition from the IFR model (columns 3-4) or by standardizing for solely the demography and assuming the same attack rate in each age group (columns 5-6). Generally, the overall IFRs were very similar between the two approaches with England as a notable exception, where the lower attack rate in the oldest age group (consistent with the seroprevalence data) drove down the IFR.

Study Location	Model Consideration	Onset to Serocon. Mean (95% CrI)	Onset to Death Mean (95% CrI)	Onset to Death Coeff. of Variation (95% CrI)	Serocon. to Serorev. Scale (95% CrI)	Serocon. to Serorev. Shape (95% CrI)
Brazil	Without Serorev.	18.3 (18.11, 18.5)	19.8 (19.61, 19.97)	0.85 (0.84, 0.86)		
Brazil	With Serorev.	18.3 (18.11, 18.5)	19.8 (19.61, 19.97)	0.85 (0.84, 0.86)	206.97 (206.85, 207)	2.95 (1.3, 3.95)
Denmark	Without Serorev.	18.3 (18.1, 18.49)	19.8 (19.61, 19.97)	0.85 (0.84, 0.86)		
Denmark	With Serorev.	18.3 (18.1, 18.49)	19.8 (19.61, 19.97)	0.85 (0.84, 0.86)	206.88 (206.36, 207)	2.76 (1.12, 3.95)
England	Without Serorev.	18.3 (18.1, 18.49)	19.79 (19.6, 19.96)	0.86 (0.85, 0.87)		
England	With Serorev.	18.3 (18.1, 18.49)	19.79 (19.6, 19.96)	0.86 (0.85, 0.87)	206.94 (206.7, 207)	3.04 (1.23, 3.96)
Italy	Without Serorev.	18.3 (18.1, 18.49)	19.81 (19.62, 19.98)	0.86 (0.84, 0.87)		
Italy	With Serorev.	18.3 (18.11, 18.49)	19.81 (19.61, 19.97)	0.86 (0.84, 0.87)	206.92 (206.6, 207)	3.9 (3.48, 4)
Netherlands	Without Serorev.	18.31 (18.11, 18.5)	19.8 (19.61, 19.97)	0.85 (0.84, 0.86)		
Netherlands	With Serorev.	18.31 (18.11, 18.51)	19.8 (19.61, 19.97)	0.85 (0.84, 0.86)	202.2 (202.01, 202.39)	2.59 (1.8, 3.46)
Spain	Without Serorev.	18.29 (18.09, 18.49)	19.79 (19.6, 19.96)	0.84 (0.83, 0.85)		
Spain	With Serorev.	18.3 (18.1, 18.5)	19.79 (19.59, 19.95)	0.84 (0.83, 0.85)	206.13 (202.45, 206.97)	3.11 (2.05, 3.95)
Sweden	Without Serorev.	18.3 (18.11, 18.5)	19.79 (19.6, 19.97)	0.85 (0.84, 0.86)		
Sweden	With Serorev.	18.31 (18.11, 18.5)	19.79 (19.6, 19.97)	0.85 (0.84, 0.86)	202.2 (202, 202.4)	2.62 (1.82, 3.5)
Geneva, Switzerland	Without Serorev.	18.3 (18.1, 18.5)	19.77 (19.58, 19.95)	0.85 (0.83, 0.86)		
Geneva, Switzerland	With Serorev.	18.3 (18.11, 18.5)	19.77 (19.57, 19.95)	0.85 (0.83, 0.86)	202.2 (202, 202.4)	2.44 (1.56, 3.37)
Zurich, Switzerland	Without Serorev.	18.3 (18.1, 18.5)	19.79 (19.59, 19.96)	0.85 (0.84, 0.86)		
Zurich, Switzerland	With Serorev.	18.3 (18.1, 18.49)	19.79 (19.59, 19.96)	0.85 (0.84, 0.86)	202.2 (202, 202.4)	2.41 (1.54, 3.35)
New York State, USA	Without Serorev.	18.3 (18.1, 18.49)	19.8 (19.6, 19.97)	0.87 (0.86, 0.88)		
New York State, USA	With Serorev.	18.3 (18.1, 18.5)	19.8 (19.6, 19.97)	0.87 (0.86, 0.88)	206.8 (205.91, 206.99)	2.85 (1.17, 3.95)

Supplementary Table 4 - Delay Distribution Posteriors: The posterior 95% credible intervals (CrI) for the mean delay from infection onset to seroconversion (Serocon.) and death as well as the coefficient of variation (Coeff. of Variation) for the onset of infection to death gamma distribution are shown for each study when seroversion was not and was considered. Similarly, the scale and shape of the onset from seroconversion to seroreversion (Serorev.) posterior distribution is given for fits where seroreversion was modelled.

Study Location	Regional Re-estimation of Sensitivity (95% CrI)	Regional Re-estimation of Specificity (95% CrI)	Age-Specific Posterior Sensitivity without Seroreversion (95% CrI)	Age-Specific Posterior Specificity without Seroreversion (95% CrI)	Age-Specific Posterior Sensitivity with Seroreversion (95% CrI)	Age-Specific Posterior Sensitivity with Seroreversion (95% CrI)
Brazil	85.14 (81.93, 87.97)	99.72 (99.55, 99.85)	85.28 (82.12, 88.14)	99.76 (99.62, 99.87)	85.27 (82.13, 88.03)	99.76 (99.62, 99.87)
Denmark	82.09 (75.51, 87.58)	99.25 (98.94, 99.56)	82.45 (76.11, 87.8)	99.16 (98.73, 99.46)	82.44 (75.94, 87.81)	99.16 (98.73, 99.47)
England	78.4 (65.68, 88.15)	99.44 (99.11, 99.71)	79.48 (68.74, 88.93)	99.59 (99.34, 99.78)	79.42 (67.58, 88.74)	99.59 (99.34, 99.78)
Italy	96.04 (89.84, 99.05)	99.7 (99.59, 99.79)	96.42 (90.93, 99.13)	99.69 (99.57, 99.78)	96.51 (90.81, 99.15)	99.69 (99.57, 99.78)
Netherlands			98.23 (95.61, 99.52)	99.83 (99.43, 99.98)	98.22 (95.55, 99.52)	99.84 (99.46, 99.98)
Spain	81.84 (75.67, 87.01)	98.79 (98.55, 99.02)	84.72 (83.08, 88.36)	99.05 (98.86, 99.21)	84.77 (83.08, 88.52)	99.05 (98.86, 99.22)
Sweden			99.28 (97.23, 99.93)	99.17 (98.12, 99.77)	99.29 (97.19, 99.94)	99.14 (98, 99.77)
Geneva, Switzerland			91.47 (87, 94.89)	99.89 (98.82, 100)	91.47 (87.05, 94.88)	99.89 (98.79, 100)
Zurich, Switzerland			91.77 (83.39, 96.89)	99.87 (99.74, 99.95)	91.73 (83.25, 96.91)	99.87 (99.75, 99.95)
New York State, USA	89.39 (85.57, 92.55)	98.73 (98.15, 99.27)	89.66 (85.9, 92.68)	98.7 (98.05, 99.2)	89.69 (86, 92.69)	98.7 (98.01, 99.19)

Supplementary table 5 - Observed serovalidation results for each included studies and posterior estimates of sensitivity and specificity: For each study, the number of test positives versus true positives reported were used to parameterize the sensitivity beta-distributed prior in the IFR model. Similarly, the number of test negatives versus true negatives reported were used to parameterize the specificity beta-distributed prior in the age-specific IFR model (Table 1, main text). For a subset of studies (6/10), regional information was used to re-estimate the true study sensitivity and specificity (using the region-based model described below). The resulting posterior medians and 95% credible intervals by study and model (without and with seroreversion) are provided for the initial simpler region-based model and the subsequent main statistical model.

Posterior predictive checks

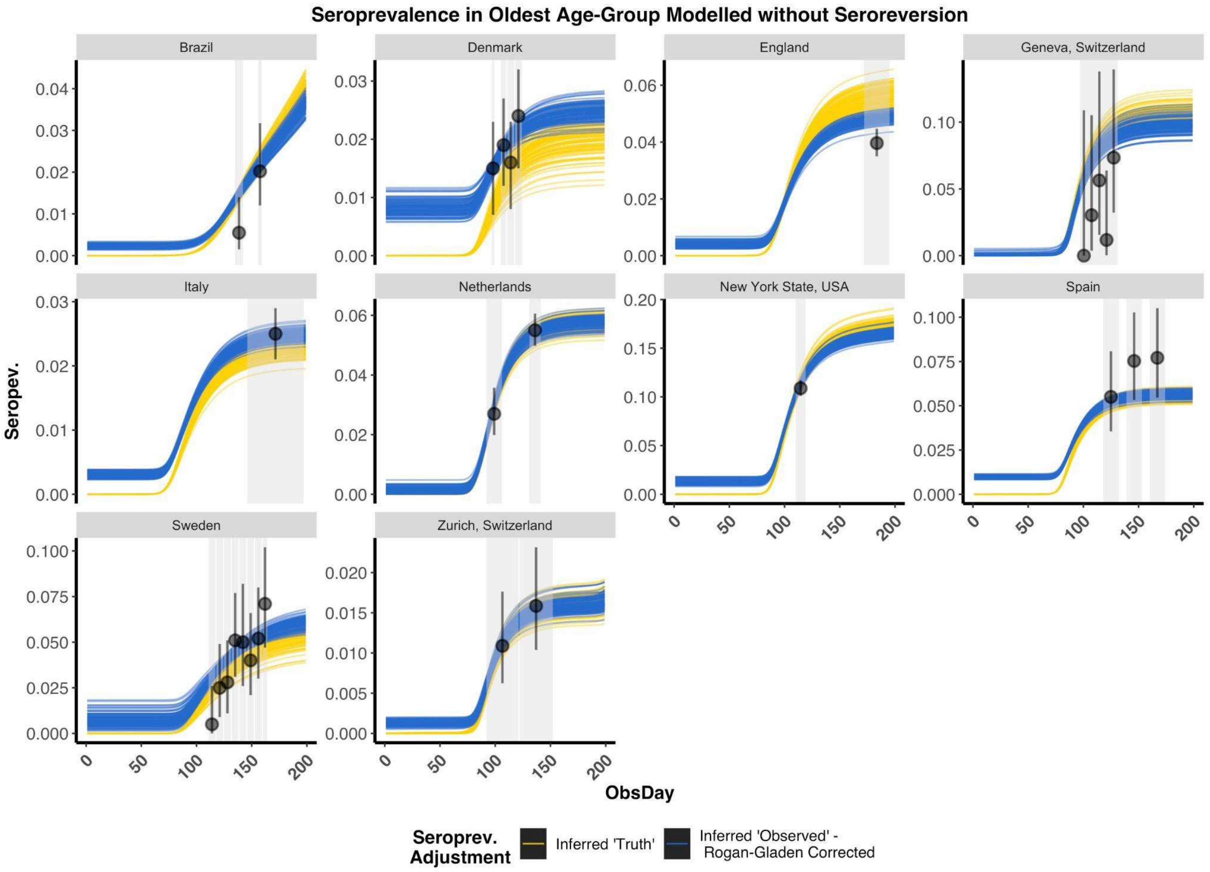

Supplementary Figure 3 - Posterior Draws of Modelled Seroprevalence without Seroreversion for the Oldest age group: Results are presented from the statistical modelling framework where seroreversion was not included. For each study and its associated model fits, we selected 100 posterior draws of the modelled true seroprevelance (yellow) based on their posterior probabilities. Due to space constraints, we only show here the results for the oldest age group, although younger age groups showed similar results. Given this true inferred seroprevalence, the expected observed seroprevalence, with the Rogan-Gladen correction, is also displayed (blue). The observed seroprevalence for the oldest age group is indicated along with its 95% confidence interval in black with the serostudy time-period demarcated by a grey box. Overall, observed seroprevalences matched closely with modelled seroprevalence and varied in some instances due to the modelled differences in the distributions of infections across age groups (Supplementary Data 1).

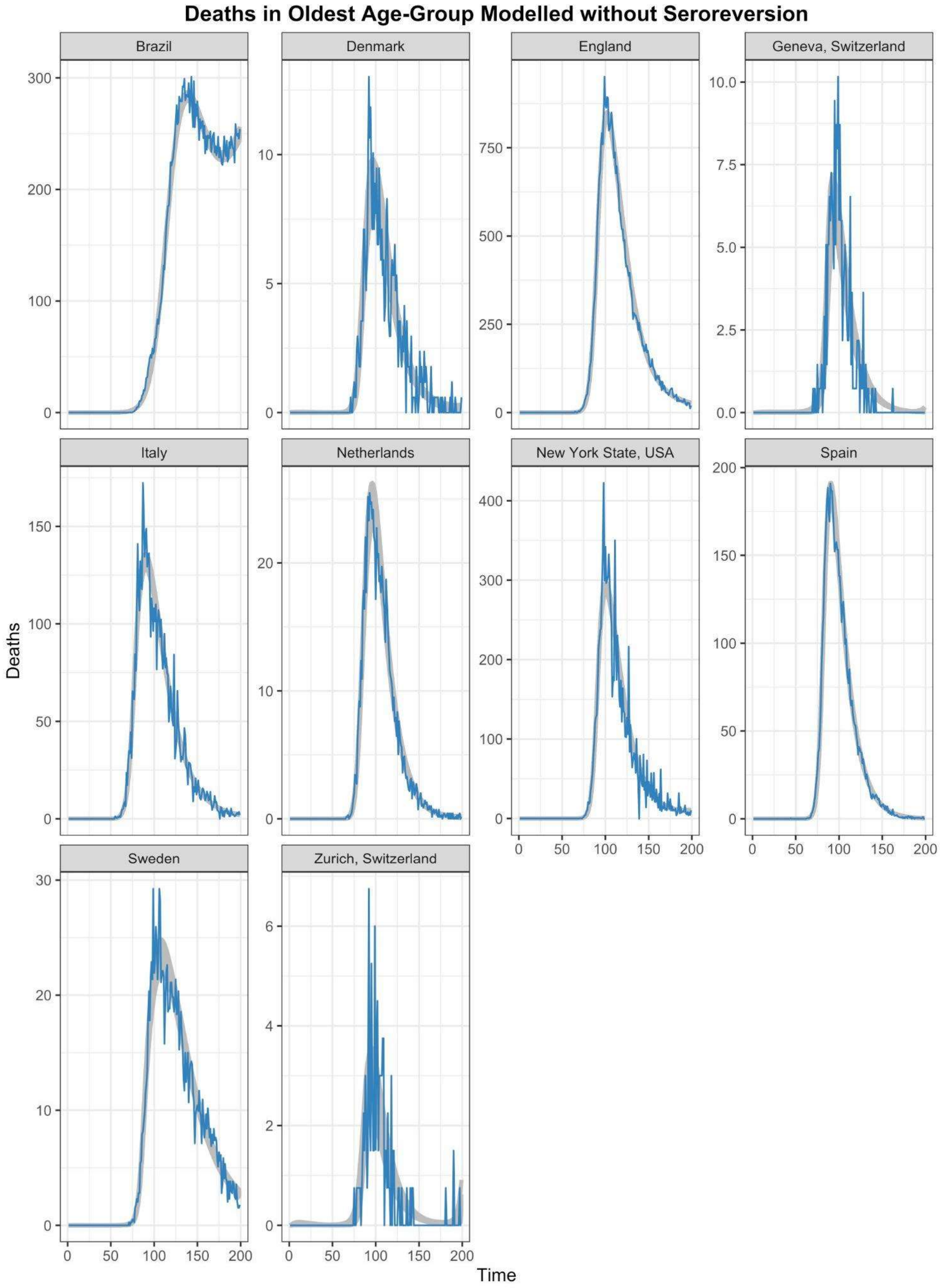

Supplementary Figure 4 - Posterior Predictive Check: Inferred Deaths without Seroreversion for the Oldest age group: Results are presented from the statistical modelling framework where seroreversion was not included. For each study and its associated model fits we selected 100 posterior draws of the modelled IFR and delay of infection onset to death based on the posterior probabilities (grey). Inferred deaths were projected forward in time according to the gamma distribution fit and compared to the age-specific time-series deaths (*i.e.* model input: blue). Due to space constraints, we only show here the results for the oldest age group, although younger age groups showed similar results.

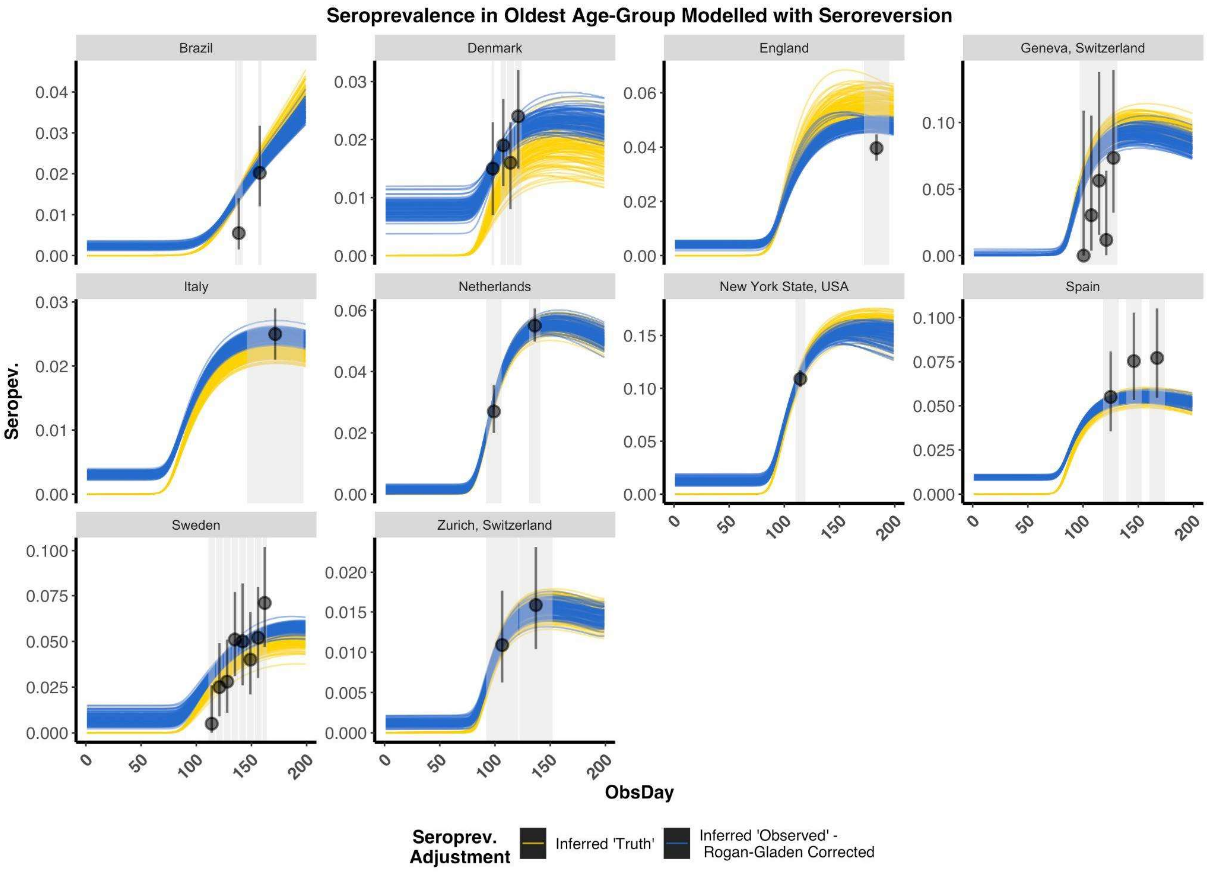

Supplementary Figure 5 - Posterior Draws of Modelled Seroprevalence with Seroreversion for the Oldest age group: Results are presented from the statistical modelling framework where seroreversion was explicitly included. The seroreversion sensitivity analysis was used to present a conservative estimate of the IFR, as we assumed a fast rate of antibody waning from the Abbott assay (see main text). For each study and its associated model fits, we selected 100 posterior draws of the modelled true seroprevelance (yellow) according to the posterior probabilities. Due to space constraints, we only show here the results for the oldest age group, although younger age groups showed similar results. Given the true inferred seroprevalence, the expected observed seroprevalence, with the Rogan-Gladen correction, is also displayed (blue). The observed seroprevalence for the oldest age group is indicated along with its 95% confidence interval in black with the serostudy time-period demarcated by a grey box. Overall, observed seroprevalences matched closely with modelled seroprevalence and varied in some instances due to the modelled differences in the distributions of infections across age groups (Supplementary Data 1). The effect of seroreversion later in the pandemic is apparent, particularly in Italy.

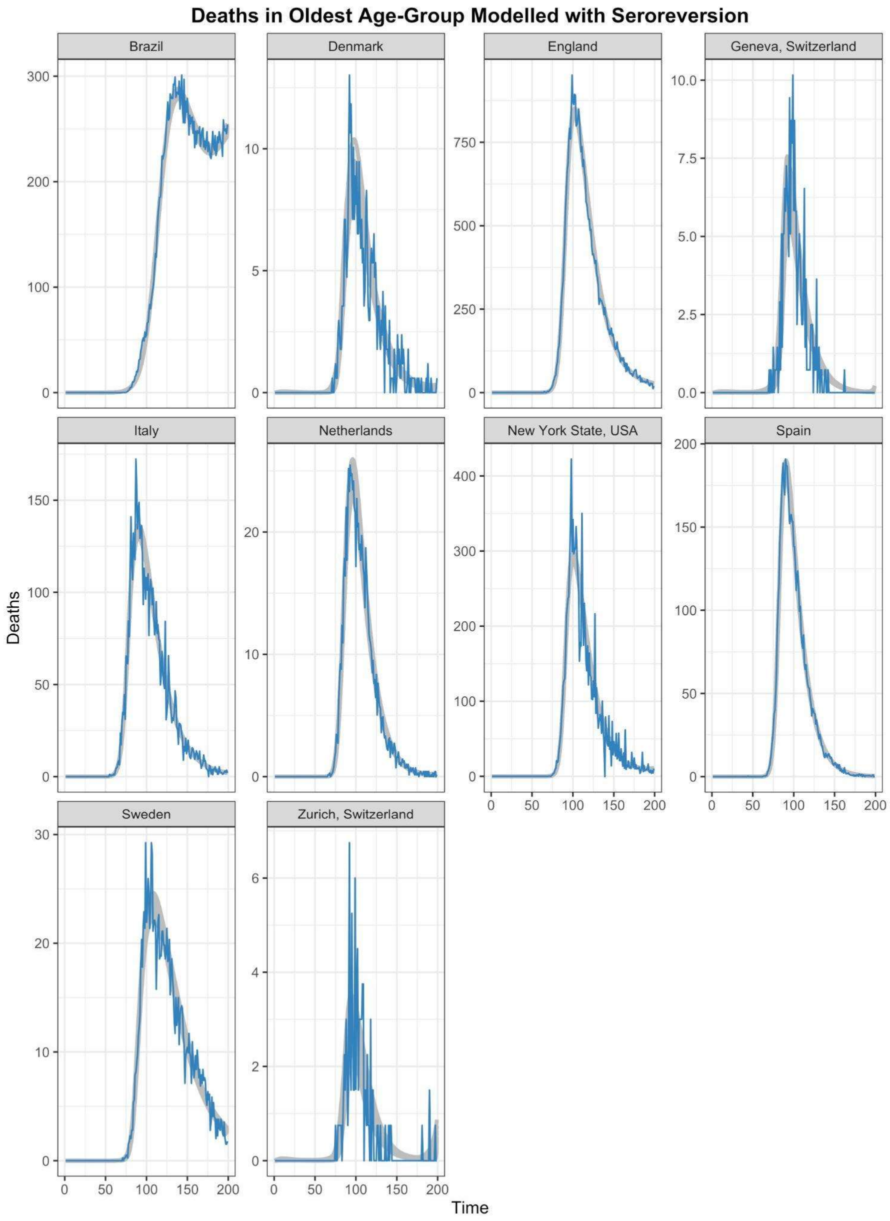

Supplementary Figure 6 - Posterior Predictive Check: Inferred Deaths with Seroreversion for the Oldest age group: Results are presented from the statistical modelling framework where seroreversion was explicitly included. The seroreversion sensitivity analysis was used to present a conservative estimate of the IFR, as we assumed a fast rate of antibody waning from the Abbott assay (see main text). For each study and its associated model fits, we selected 100 posterior draws of the modelled IFR and delay of infection onset to death based on the posterior probabilities (grey). Inferred deaths were projected forward in time according to the gamma distribution fit and compared to the recasted age-specific time-series deaths (*i.e.* model input: blue). Due to space constraints, we only show here the results for the oldest age group, although younger age groups showed similar results.

Specificity estimates from regional data

Supplementary Figure 7 - Regional model fits to data for six studies with regional serosurvey data: For each country: (A) relationship between seroprevalence and COVID-19 mortality per 100,000 population in the data (observed seroprevalence not adjusted for test sensitivity and specificity) and model-estimated true seroprevalence (adjusted for test sensitivity and specificity) and mortality with 95% credible intervals. (B) as A but showing model-estimated observed seroprevalence (as would be observed given the model-estimated sensitivity and specificity). (C) Specificity posterior distribution (solid line) versus the value reported in the study (dashed line) (D) Observed versus model-estimated age-specific seroprevalence (adjusted for sensitivity and specificity).

Spain

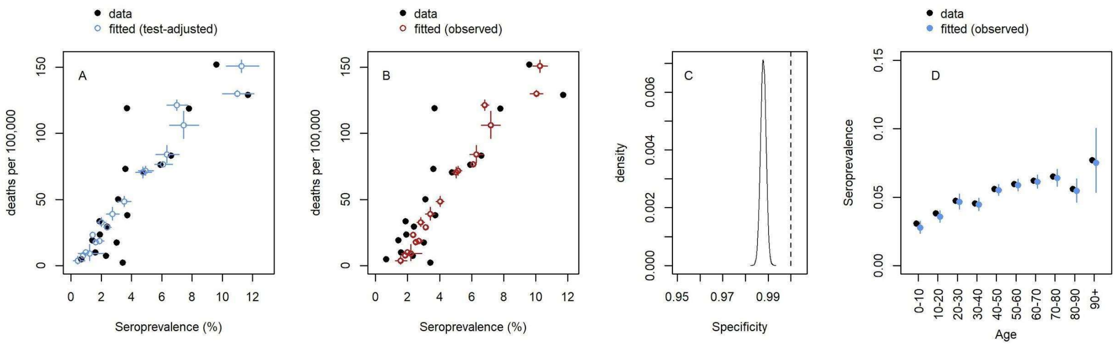

Italy

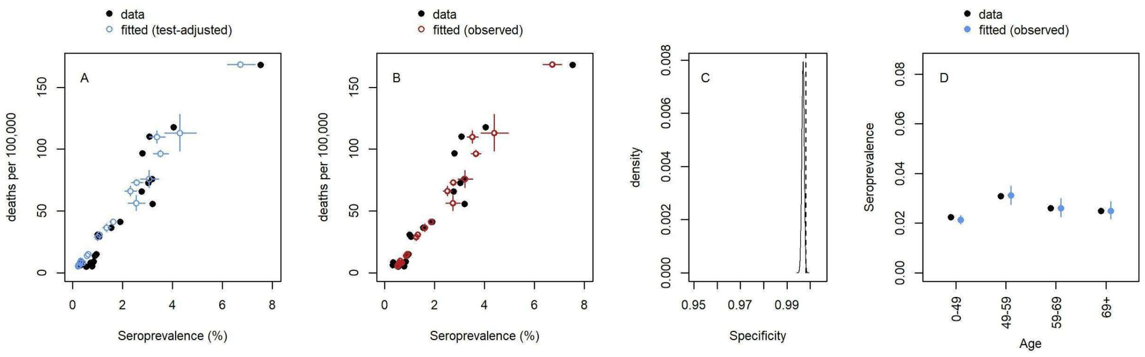

Denmark (average seroprevalence across all ages assumed to be the same due to lack of overlap of mortality and seroprevalence data age groups)

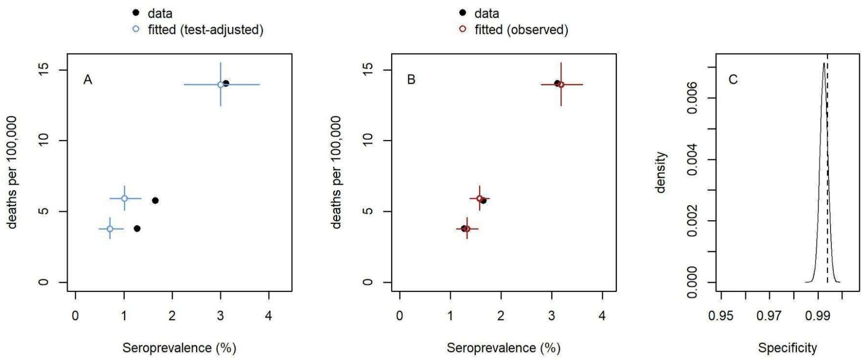

New York State

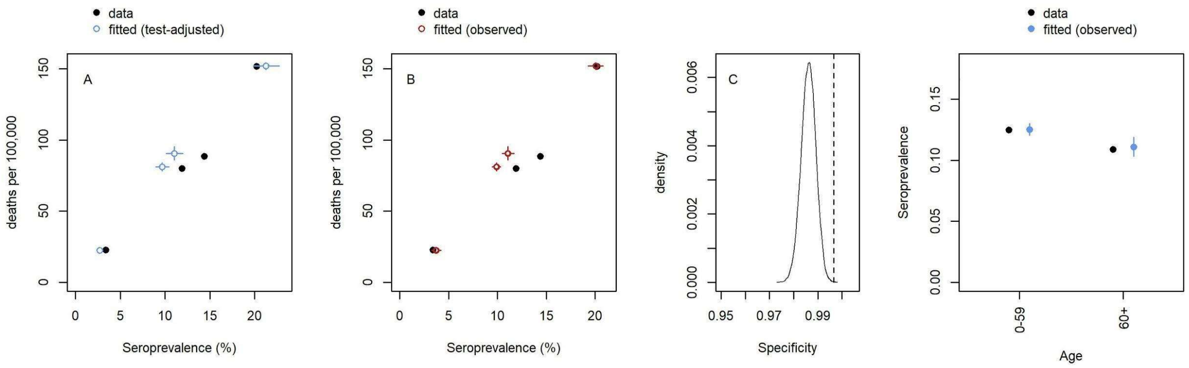

England

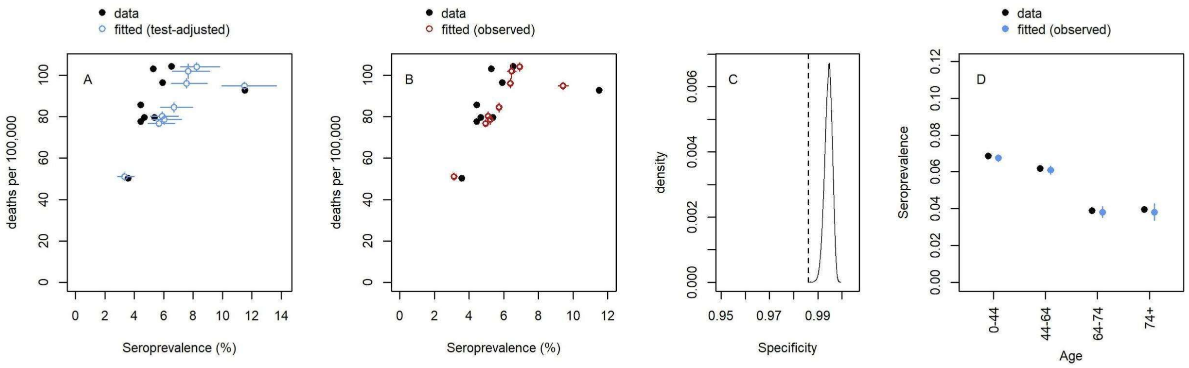

Brazil

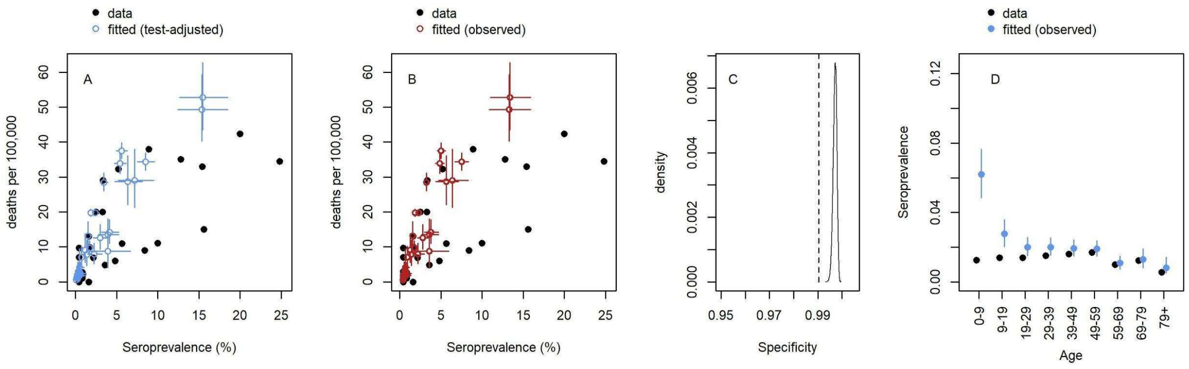

Supplementary Note 4. Pooled IFR calculation

We pooled the posterior age-specific IFR estimates across studies using weighted log-linear regression, where weights were calculated as the posterior precision of age- and study-specific IFR estimates. After fitting the weighted log-linear regression model, we found that there was heteroskedasticity in the model residuals (**Supplementary Figure 8**). To account for this, we modelled variance as a quadratic equation with respect to the mean age within each age group for each study categorized into one of the 10-year age bands (**Table 3**). Our final model therefore consists of a fitted linear model for the mean of the log IFR by age, with the variance in log IFR changing with age. Under the assumption of normally distributed residuals in log space, this corresponds to a log-normal model in linear space, in which the mean is a function of both the mean and the variance in log IFR. This log-normal model was used to produce the final predictive intervals in 5-year age bands.

We then standardized these age-specific estimates to the population demographies of four countries: Madagascar, Nicaragua, Grenada and Malta, which have previously been identified as representative countries for each of the World Bank Income Strata: LIC, LMIC, UMIC, and HIC[15]. The overall IFR prediction interval was estimated using a Monte Carlo sampling approach: repeatedly drawing from the appropriate log-normal distribution (as described above) and combining values as weighted sums, where weights were calculated from the age-specific demographics. Pooled log linear models fit with demographic weights by age groups resulted in similar age-specific pooled IFR estimates (not shown).

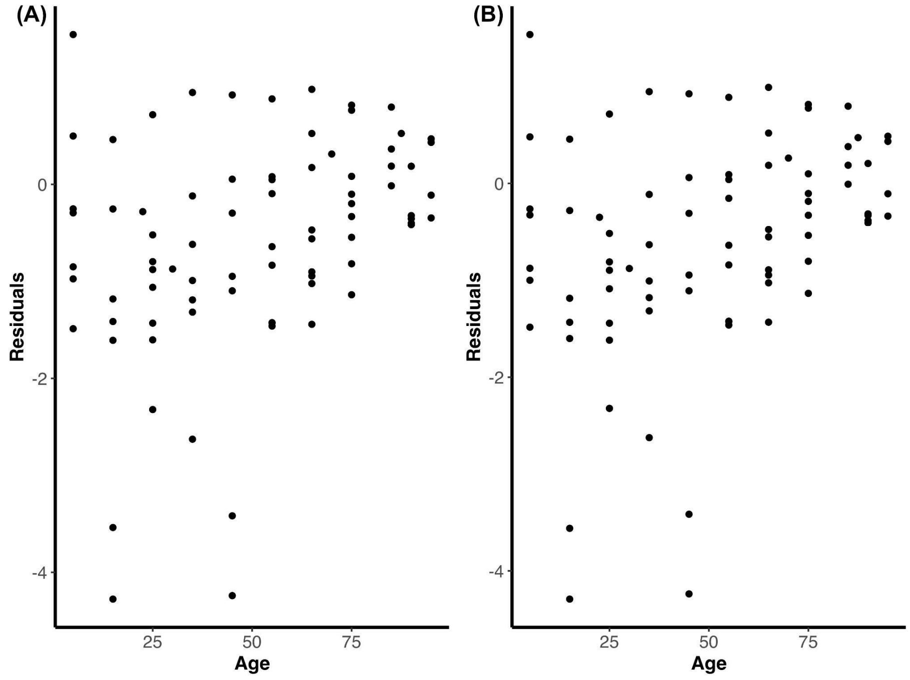

Supplementary Figure 8 - Weight Log-Linear Model Residuals: The model residuals from the weighted log-linear pooled fits are shown when seroreversion was not considered (**A**) and was considered (**B**). The residuals show heteroscedasticity, particularly among the middle age groups.

Age	Variance from Modelled Estimates without Seroreversion	Variance from Modelled Estimates with Seroreversion
5	1.0297	1.0304
15	2.8544	2.8631
25	0.7456	0.7398
35	1.0441	1.0425
45	3.5077	3.5048
55	0.6823	0.6789
65	0.6415	0.643
75	0.4042	0.3983
85	0.096	0.0915
95	0.131	0.1302

Supplementary table 6 - Variance within the 10-year age groups for the Pooled IFR estimates Log-Linear Model Residuals: The corresponding variance for the 10-year age groups calculated from the modelled residuals and used in the pooled IFR calculations.

Supplementary References

1. Muecksch, F. *et al.* Longitudinal Serological Analysis and Neutralizing Antibody Levels in Coronavirus Disease 2019 Convalescent Patients. *J. Infect. Dis.* **223**, 389–398 (2021).
2. Therneau, T. M. & Grambsch, P. M. *Modeling Survival Data: Extending the Cox Model.* (Springer Science & Business Media, 2000).
3. Therneau, T. M. *A Package for Survival Analysis in R*. https://CRAN.R-project.org/package=survival (2020).
4. Borremans, B. *et al.* Quantifying antibody kinetics and RNA shedding during early-phase SARS-CoV-2 infection. *medRxiv* (2020).
5. Knock, E. S. *et al.* The 2020 SARS-CoV-2 epidemic in England: key epidemiological drivers and impact of interventions. *bioRxiv* (2021) doi:10.1101/2021.01.11.21249564.
6. Borremans, B. *et al.* Quantifying antibody kinetics and RNA detection during early-phase SARS-CoV-2 infection by time since symptom onset. *Elife* **9**, (2020).
7. Li, Q. *et al.* Early Transmission Dynamics in Wuhan, China, of Novel Coronavirus–Infected Pneumonia. *N. Engl. J. Med.* **382**, 1199–1207 (2020).
8. Linton, N. M. *et al.* Incubation Period and Other Epidemiological Characteristics of 2019 Novel Coronavirus Infections with Right Truncation: A Statistical Analysis of Publicly Available Case Data. *J. Clin. Med. Res.* **9**, (2020).
9. Verity, R. *et al.* Estimates of the severity of coronavirus disease 2019: a model-based analysis. *Lancet Infect. Dis.* (2020) doi:10.1016/S1473-3099(20)30243-7.
10. Wood, S. N., Wit, E. C., Fasiolo, M. & Green, P. J. COVID-19 and the difficulty of inferring epidemiological parameters from clinical data. *Lancet Infect. Dis.* (2020) doi:10.1016/S1473-3099(20)30437-0.
11. Salje, H. *et al.* Estimating the burden of SARS-CoV-2 in France. *Science* **369**, 208–211 (2020).
12. Delignette-Muller, M. L. & Dutang, C. fitdistrplus: An R Package for Fitting Distributions. *Journal of Statistical Software* vol. 64 1–34 (2015).
13. Carpenter, B. *et al.* Stan: A probabilistic programming language. *J. Stat. Softw.* **76**, (2017).
14. Arora, R. K. *et al.* SeroTracker: a global SARS-CoV-2 seroprevalence dashboard. *Lancet Infect. Dis.* (2020) doi:10.1016/S1473-3099(20)30631-9.
15. Walker, P. G. T. *et al.* The impact of COVID-19 and strategies for mitigation and suppression in low- and middle-income countries. *Science* (2020) doi:10.1126/science.abc0035.
16. United Nations, Department of Economic and Social Affairs Population Dynamics. World Population Prospects 2019. https://population.un.org/wpp.
17. Hallal, P. C. *et al.* SARS-CoV-2 antibody prevalence in Brazil: results from two successive nationwide serological household surveys. *Lancet Glob Health* **8**, e1390–e1398 (2020).
18. Silveira, M. F. *et al.* Population-based surveys of antibodies against SARS-CoV-2 in Southern Brazil. *Nat. Med.* **26**, 1196–1199 (2020).
19. SRAG 2020 - Severe Acute Respiratory Syndrome Database - including data from COVID-19. https://opendatasus.saude.gov.br/dataset/bd-srag-2020. Accessed on June 15, 2020.
20. Geografi, I. B. Projeções da População. https://www.ibge.gov.br/estatisticas/sociais/populacao/9109-projecao-da-populacao.html?=&t=resultados. Accessed on June 15, 2020.
21. Erikstrup, C. *et al.* Estimation of SARS-CoV-2 infection fatality rate by real-time antibody screening of blood donors. *Clin. Infect. Dis.* (2020) doi:10.1093/cid/ciaa849.
22. Coronavirus: Som bloddonor hjælper du nu med at afdække det danske mørketal - Bloddonorerne i Danmark. https://bloddonor.dk/coronavirus/. Accessed on Sep 28, 2020.
23. Tal og overvågning: COVID-19. https://www.sst.dk/da/corona/tal-og-overvaagning.

Accessed on Oct 19, 2020.
24. Statens Serum Institute, Denmark. Overvågning af COVID-19. https://www.ssi.dk/sygdomme-beredskab-og-forskning/sygdomsovervaagning/c/covid19-overvaagning. Accessed on Oct 19, 2020.
25. Statistik, D. Statistikbanken. https://www.statistikbanken.dk/statbank5a/. Accessed on June 6, 2020. (2011).
26. Flower, B. *et al.* Clinical and laboratory evaluation of SARS-CoV-2 lateral flow assays for use in a national COVID-19 seroprevalence survey. *Thorax* (2020) doi:10.1136/thoraxjnl-2020-215732.
27. Ward, H. *et al.* SARS-CoV-2 antibody prevalence in England following the first peak of the pandemic. *Nat. Commun.* **12**, 905 (2021).
28. Excess mortality in England, week ending 17 July 2020. https://fingertips.phe.org.uk/static-reports/mortality-surveillance/excess-mortality-in-england-week-ending-17-Jul-2020.html. Accessed on July 17, 2020.
29. Deaths registered weekly in England and Wales, provisional. https://www.ons.gov.uk/peoplepopulationandcommunity/birthsdeathsandmarriages/deaths/datasets/weeklyprovisionalfiguresondeathsregisteredinenglandandwales. Accessed on July 17, 2020.
30. Population Estimates. https://www.ons.gov.uk/peoplepopulationandcommunity/populationandmigration/populationestimates. Accessed on July 2, 2020.
31. Primi Risultati Dell'indagine Di Sieroprevalenza Sul SARS-CoV-2. https://www.istat.it/it/files//2020/08/ReportPrimiRisultatiIndagineSiero.pdf. Accessed 14 Aug 2020.
32. Ng, D. L. *et al.* SARS-CoV-2 seroprevalence and neutralizing activity in donor and patient blood from the San Francisco Bay Area. *medRxiv* (2020) doi:10.1101/2020.05.19.20107482.
33. Demographics of COVID-19 Deaths. https://dc-covid.site.ined.fr/en/ Accessed 19th Oct 2020.
34. Italian Ministry of Health. http://www.salute.gov.it/portale/nuovocoronavirus/. Accessed on Oct 19, 2020.
35. Resident population by age, sex and marital status and Resident Foreigners by age and sex. https://www.istat.it/en/archivio/132669. Accessed on Aug 6, 2020. (2014).
36. Slot, E. *et al.* Herd immunity is not a realistic exit strategy during a COVID-19 outbreak. *ResearchSquare Preprint* (2020).
37. Study shows coronavirus antibodies in 5.5% of Dutch blood donors. *Reuters* (2020).
38. Covid-19 karakteristieken per casus landelijk. https://data.rivm.nl/meta/srv/eng/catalog.search#/metadata/2c4357c8-76e4-4662-9574-1deb8a73f724?tab=relations. Accessed on June 3, 2020.
39. National Institute for Public Health and the Environment & Ministry of Health, Welfare and Sport, the Netherlands. Development of COVID-19 in Graphs. https://www.rivm.nl/coronavirus-covid-19/grafieken. Accessed on Oct 19, 2020.
40. CBS Dataportaal. https://opendata.cbs.nl/statline/portal.html?_la=en&_catalog=CBS&tableId=03743eng&_theme=1056. Accessed June 11 2020.
41. Pollán, M. *et al.* Prevalence of SARS-CoV-2 in Spain (ENE-COVID): a nationwide, population-based seroepidemiological study. *Lancet* **396**, 535–544 (2020).
42. Ministerio de Sanidad, Consumo y Bienestar Social - Ciudadanos - Estudio Nacional de sero-Epidemiología de la Infección por SARS-CoV-2 en España (ENE-Covid). https://www.mscbs.gob.es/ciudadanos/ene-covid/. Accessed on Sep 11, 2020.
43. National Institute of Demographic Studies (INED). Spanish Ministry of Health (Data

obtained from the database The demography of deaths by COVID-19). https://dc-covid.site.ined.fr/fr/. Accessed Sep 11, 2020.
44. INE. Instituto Nacional de Estadistica. https://www.ine.es/. Accessed on May 19, 2020.
45. Sweden Public Health Agency. Påvisning av antikroppar efter genomgången covid-19 i blodprov från öppenvården (Delrapport 1). https://www.folkhalsomyndigheten.se/contentassets/9c5893f84bd049e691562b9eeb0ca280/pavisning-antikroppar-genomgangen-covid-19-blodprov-oppenvarden-delrapport-1.pdf. Accessed on Oct 21, 2020.
46. Sweden Public Health Agency. Påvisning av antikroppar efter genomgången covid-19 hos blodgivare (Delrapport 2). https://www.folkhalsomyndigheten.se/contentassets/376f9021a4c84da08de18ac597284f0c/pavisning-antikroppar-genomgangen-covid-19-blodgivare-delrapport-2.pdf. Accessed on Oct 21, 2020.
47. Sweden Public Health Agency. Bekräftade fall i Sverige – daglig uppdatering. https://www.folkhalsomyndigheten.se/smittskydd-beredskap/utbrott/aktuella-utbrott/covid-19/bekraftade-fall-i-sverige/. Accessed on June 4, 2020.
48. Folkmängden efter region, civilstånd, ålder och kön. År 1968 - 2019. http://www.statistikdatabasen.scb.se/pxweb/sv/ssd/START__BE__BE0101__BE0101A/BefolkningNy/?rxid=938858cd-aeaf-4a53-80de-7b060f869878. Accessed on Aug 8, 2020.
49. Meyer, B. *et al.* Validation of a commercially available SARS-CoV-2 serological immunoassay. *Clin. Microbiol. Infect.* **26**, 1386–1394 (2020).
50. Stringhini, S. *et al.* Seroprevalence of anti-SARS-CoV-2 IgG antibodies in Geneva, Switzerland (SEROCoV-POP): a population-based study. *Lancet* **396**, 313–319 (2020).
51. Federal Office of Public Health. COVID-19 en Suisse. https://covid-19-schweiz.bagapps.ch/fr-1.html. Accessed on Sep 29, 2020.
52. Federal Office of Public Health. Situation in Switzerland. https://www.bag.admin.ch/bag/en/home/krankheiten/ausbrueche-epidemien-pandemien/aktuelle-ausbrueche-epidemien/novel-cov/situation-schweiz-und-international.html. Accessed Sep 29, 2020.
53. Emmenegger, M. *et al.* Early plateau of SARS-CoV-2 seroprevalence identified by tripartite immunoassay in a large population. *medRxiv* (2020).
54. Rosenberg, E. S. *et al.* Cumulative incidence and diagnosis of SARS-CoV-2 infection in New York. *Ann. Epidemiol.* **48**, 23–29.e4 (2020).
55. New York State Department of Health. Fatalites. https://covid19tracker.health.ny.gov/views/NYS-COVID19-Tracker/NYSDOHCOVID-19Tracker-Fatalities. Accessed on Sep 3, 2020.
56. Dong, E., Du, H. & Gardner, L. An interactive web-based dashboard to track COVID-19 in real time. *Lancet Infect. Dis.* **20**, 533–534 (2020).
57. COVID-19 Data Repository by the Center for Systems Science and Engineering (CSSE) at Johns Hopkins University. Accessed Nov 2020.
58. United States Census Bureau. County Population by Characteristics: 2010-2019. https://www.census.gov/data/tables/time-series/demo/popest/2010s-counties-detail.html. Accessed on June 20, 2020.
