# Supplementary material for: Estimating the COVID-19 infection fatality ratio accounting for seroreversion using statistical modelling
Source: Commun Med (Lond). 2022 May 19;2:54. doi: 10.1038/s43856-022-00106-7 (PMC9120146; doi:10.1038/s43856-022-00106-7)
Supplement: Supplementary file 3 — Description of Additional Supplementary Files [file 43856_2022_106_MOESM3_ESM.pdf]

Description of Additional Supplementary Files

File Name: Supplementary Data 1

Description: Age Specific Estimates of the Infection Fatality Ratio across Included Studies: The age-specific IFR estimates are provided with 95% credible intervals (CrI) for models where seroversion was not and was considered. The crude IFRs and 95% confidence intervals (CI) are included for comparison. In addition, the inferred observed seroprevalence -- from 100 posterior draws based on the posterior probabilities and corrected for test sensitivity and specificity with the Rogan-Gladen equation -- is provided alongside the observed seroprevalence for the latest serosurvey with respect to each study and age group. Ages of “999” indicate an upper bound.
